# Triazole-imidazo[1,2-*b*]pyrazoles Able to Counteract Melanoma Cell Survival Without Compromising the Viability of Healthy Keratinocytes

**DOI:** 10.3390/ijms26136312

**Published:** 2025-06-30

**Authors:** Chiara Brullo, Barbara Marengo, Cinzia Domenicotti, Matteo Lusardi, Elena Cichero, Annalisa Salis, Debora Caviglia, Eleonora Russo, Andrea Spallarossa

**Affiliations:** 1 Department of Pharmacy, University of Genoa, Viale Benedetto XV 3, 16132 Genova, Italy; matteo.lusardi@edu.unige.it (M.L.); elena.cichero@unige.it (E.C.); debora.caviglia@edu.unige.it (D.C.); eleonora.russo@unige.it (E.R.); andrea.spallarossa@unige.it (A.S.); 2 Department of Experimental Medicine (DIMES), University of Genova, Via Leon Battista Alberti 2, 16132 Genoa, Italy; barbara.marengo@unige.it (B.M.); cinzia.domenicotti@unige.it (C.D.); 3 IRCCS Ospedale Policlinico San Martino, Largo Rosanna Benzi 10, 16132 Genoa, Italy; 4 Centro 3R, Department of Information Engineering, University of Pisa, Largo Lucio Lazzarino 1, 56122 Pisa, Italy; 5 Department of Experimental Medicines, University of Genoa, Viale Benedetto XV 1, 16132 Genoa, Italy; annalisa.salis@unige.it

**Keywords:** imidazo-pyrazole derivatives, anti-proliferative agents, melanoma cells, drug delivery, critical micelle concentration, surfactants

## Abstract

To further extend the structure–activity relationships on previously identified anti-proliferative imidazo-pyrazoles, a novel series of compounds was designed and synthesized. In the obtained derivatives (**1**), the imidazo-pyrazole scaffold was formally condensed with a substituted triazole moiety, known for its biological properties. All derivatives were tested for anti-proliferative activity on a panel of 60 different cancer cell lines and compound **1h** was identified as the most promising derivative, being highly effective against melanoma cells. Additional investigations demonstrated a cytotoxic and pro-oxidant action of the compound **1h** on human metastatic melanoma cell lines (MeOV and MeTA) but not on healthy keratinocytes (HaCAT), confirming the selective activity of the compound. In silico calculations predicted favorable drug-like and pharmacokinetic properties and pre-formulation studies evaluated the effect of Tween 80 on **1h** solubility. Overall, the collected data confirmed the pharmacological potential of the imidazo-pyrazole scaffold and indicated **1h** as an interesting lead structure for the development of novel anti-melanoma agents.

## 1. Introduction

The 1,2,4-triazole nucleus, unfused or linked to other nitrogenous heterocycles, represents a pivotal pharmacophore, being embedded in different biologically active compounds including anticonvulsants [1], fungicides [2], herbicides [3], antibacterials [4], antigastrics [5], anti-oxidants [6], modulators or antagonists of ion channels [7], agents effective on metabolic syndrome (e.g., GPR131 and 11β hydroxysteroid dehydrogenase inhibitors) [8,9], and D3 antagonists [10,11,12]. Moreover, 1,2,4-triazole-containing compounds showed relevant anti-cancer activity [13,14,15,16,17]; interestingly, the presence of a sulfur-containing substituent at position 3 and a nitrogen heterocycle at position 5 of the triazole ring were correlated with the most potent anti-proliferative activity [18].

**Figure 1:**
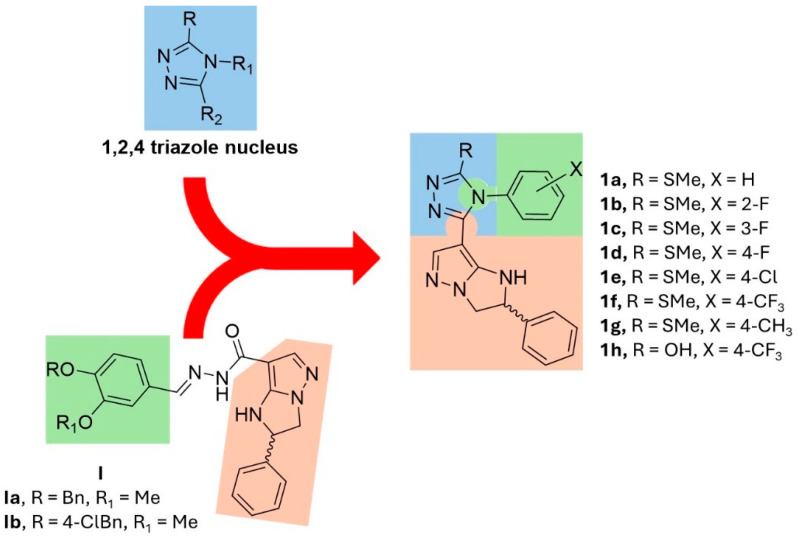
Structures of 1,2,4 triazole nucleus, previous anti-cancer imidazo-pyrazoles **I**, and new designed compounds **1a**–**h**.

**Figure 2:**
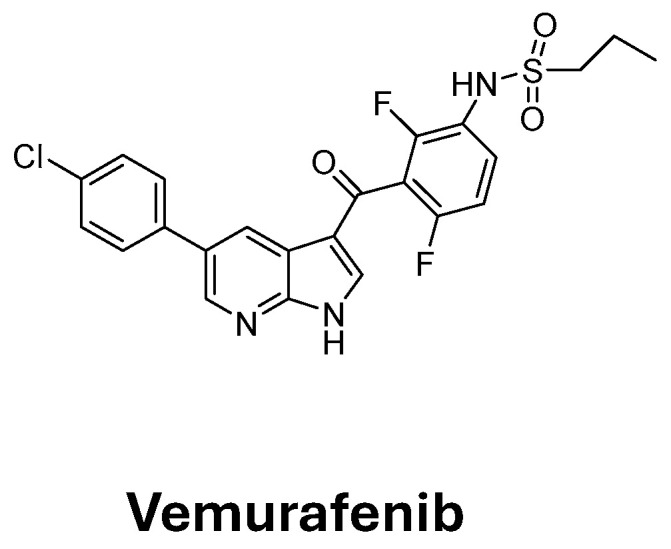
Structure of Vemurafenib.

In previous studies, we synthesized different imidazo-pyrazoles **I** (Figure 1) with interesting anti-cancer, anti-angiogenic, and anti-inflammatory activities, showing anti-proliferative activity with IC_50_ values in the low micromolar range against selected tumor cell lines [19,20]. Furthermore, derivative **Ia** proved to be effective against patient-isolated melanoma (MEOV NT) and Vemurafenib (PLX4032, Figure 2)-resistant (MEOV PLX-R) cells, representing a valuable lead compound for the development of novel therapeutics for melanoma treatment [21]. Additionally, imidazo-pyrazole derivative **Ib** (Figure 1) showed low micromolar IC_50_ values against the skin melanoma SKMEL-28 cell line [22] and proteomics studies evidenced its ability to downregulate Ras-responsive element binding protein 1 (RREB1), a crucial molecular target in SKMEL-28 melanoma cells [23]. Collectively, these studies confirmed the imidazo-pyrazole moiety as an interesting chemical scaffold to develop novel anti-melanoma agents.

To further extend the structure–activity relationships (SARs) of imidazo-pyrazoles **I** and to obtain more active anti-proliferative agents, we designed and synthesized novel compounds **1a**–**g** (Figure 1) in which the imidazo-pyrazole substructure was functionalized with a 1,2,4 triazole nucleus. In detail, (i) 1,2,4-triazole nucleus was inserted at position 7 of the imidazo-pyrazole scaffold, in analogy to the functionalization pattern of previous **I**; (ii) a substituted phenyl ring, structurally related to the catechol moiety of **I**, was inserted at position 4 of triazole; (iii) the position 3 of 1,2,4 triazole was substituted with a thiomethyl (compounds **1a**–**g**) or a hydroxy group (**1h**).

To identify potential hit compounds, derivatives **1** were initially tested for anti-proliferative activity on a panel of 60 different cancer cell lines by the National Cancer Institute (Developmental Therapeutics Program, Division of Cancer Treatment and Diagnosis). Out of this initial screening, compound **1h** was selected as the most promising analogue and submitted to further investigations. In detail, the cytotoxic and pro-oxidant effects of **1h** were evaluated in two human metastatic melanoma cell lines, namely MeOV (characterized by the most common BRAF^V600D^ mutation) and MeTA (characterized by BRAF^V600E^ mutation). Furthermore, the same evaluations were carried out on healthy HaCAT keratinocytes to verify the cytotoxicity of **1h** against non-cancerous cells. Moreover, in silico drug-like and pharmacokinetic properties were analyzed and the micellar solubilization of **1h** was performed to provide new imidazo-pyrazole-based drug delivery systems with enhanced and anti-cancer activity.

## 2. Results

### 2.1. Chemistry

**Scheme 1:**
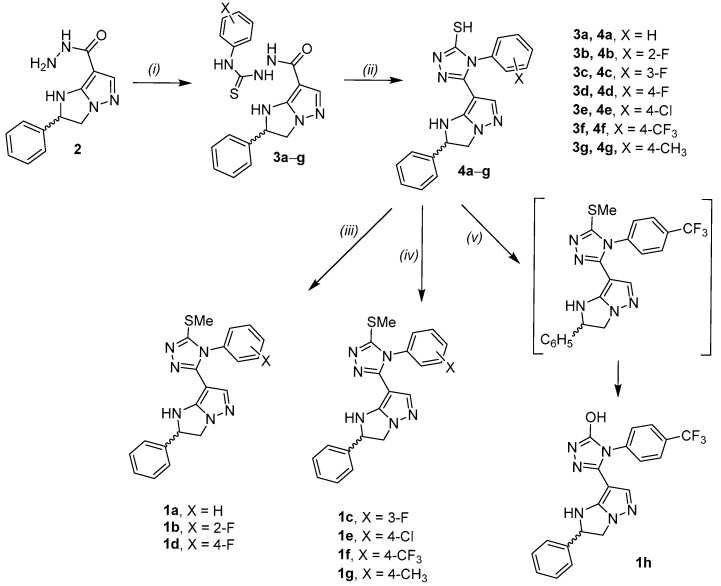
Reaction and conditions: (i) proper phenylisothiocyanate, EtOH ass., 6–18 h; (ii) 2M NaOH, 80 °C, 4–12 h; (iii) CH_3_I, AcONa, EtOH ass., reflux 3–4 h; (iv) CH_3_I, K_2_CO_3_. an. DMF, r.t., 1–18 h; (v) CH_3_I, K_2_CO_3_. an. DMF, r.t. 24 h.

Derivatives **1** were synthesized according to a sequential strategy starting from the key carbohydrazide intermediate **2** (Scheme 1) previously prepared by us following the literature procedure [20]. In particular, **2** was reacted with the suitable substituted phenyl isothiocyanates in ethanol at reflux for 6–18 h to obtain intermediates **3a**–**f** that were cyclized in a basic condition (2M NaOH) for 4–12 h to afford the 1,2,4 triazole derivatives **4** (Scheme 1). Compounds **1a**–**g** were synthesized by the methylation of the corresponding thiol precursors with iodomethane in the presence of either sodium acetate in absolute ethanol (compounds **1a**,**b**,**d**) or potassium carbonate in anhydrous DMF (compounds **1c**,**e**–**g**). Compound **1h** was prepared by a one-pot, two-step reaction by condensing **4f** with iodomethane in the presence of potassium carbonate in anhydrous DMF and prolonging stirring for 24 h.

### 2.2. Biological Results

#### 2.2.1. Anti-Proliferative Activity

New synthesized compounds **1a**–**h** were tested for anti-proliferative activity by the National Cancer Institute (Developmental Therapeutics Program, Division of Cancer Treatment and Diagnosis) at a fixed concentration of 10 µM [24]. Out of the eight compounds tested, only derivatives **1a**, **1e** and **1h** evidenced percentage growth values below ≤60% for at least one of the cell lines considered (Table 1 and Supplementary Materials Figures S31–S33). In detail, two compounds (bearing a thiomethyl substituent on a triazole scaffold) were able to inhibit the growth of leukemia and non-small-cell lung cancer (NSCLC) cells (**1a**), and melanoma and renal cancer (**1e**), whereas **1h**, characterized by a 3-hydroxy substituted triazole nucleus, strongly inhibited the growth of several cancer cell lines investigated, affecting particularly leukemia, colon cancer, and melanoma cell proliferation. Interestingly, the replacement of **1h** hydroxy group with a thiomethyl substituent completely abolished anti-cancer activity (compare **1h** and **1f**).

#### 2.2.2. **1h** Affects the Viability of MeOV and MeTA Cell Lines

**Figure 3:**
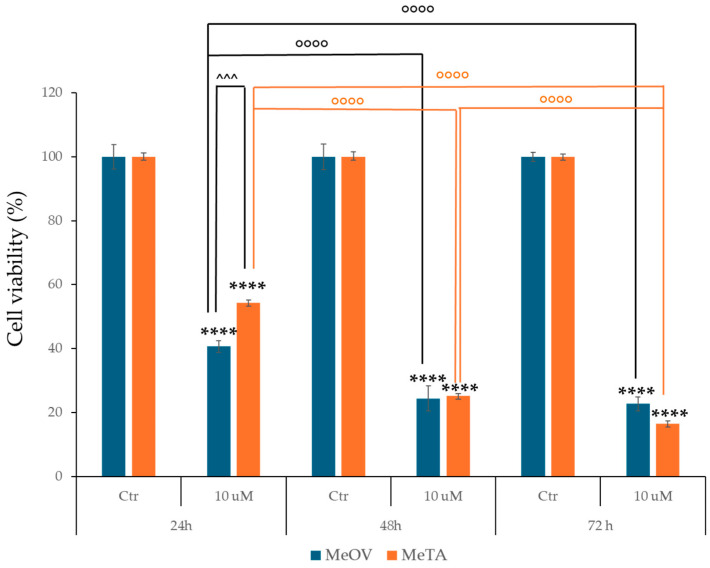
**1h** exposure reduces cell viability of melanoma cells. Cell viability was evaluated by MTS assay in MeOV (blue bars) and in MeTA cells (orange bars) treated with 10 µM **1h** for 24, 48, and 72 h. The graph reports the percentage viability of cells treated for indicated times in comparison with that of untreated ones (100%). Histograms summarize quantitative data of means ± S.E.Ms of at least four independent experiments. **** *p* < 0.0001 vs. untreated MeOV or MeTRAV cells. ◦◦◦◦ *p* < 0.0001 vs. respective treatments indicated by the bars.; ^^^ *p* < 0.001 vs. respective treatments indicated by the bars.

Based on NCI screening results, human metastatic melanoma cells MeOV and MeTA, were treated with **1h** (10 µM) for 24 h, 48 h, and 72 h. As shown in Figure 3, 24 h treatment decreased the viability of MeOV and MeTA by 60% and 46%, respectively. Incubation for 48 h and 72 h further increased the cytotoxic effect of **1h**, reducing the viability of MeOV and MeTA cells by 77% and 85%, respectively, at 72 h (Figure 3).

#### 2.2.3. **1h** Stimulates the Production of Reactive Oxygen Species (ROS) in MeOV and MeTA Cell Lines

**Figure 4:**
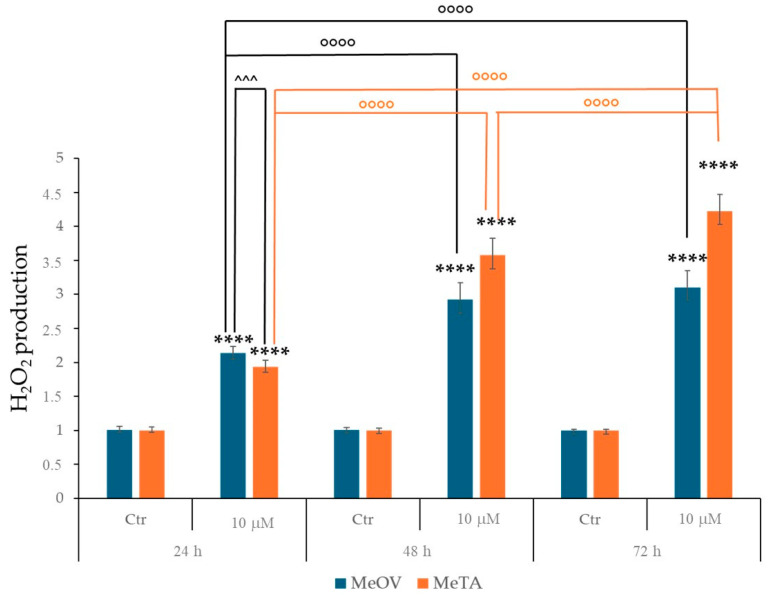
**1h** exposure enhances H_2_O_2_ production in a time-dependent way. H_2_O_2_ production was analysed by DCFH-DA labelling in MeOV (blue bars) MeTA cells (orange bars) treated with 10 µM **1h** for 24, 48, and 72 h. Results are expressed as variation of H_2_O_2_ production under treatment conditions in comparison with untreated ones (1). Histograms summarize quantitative data of means ± S.E.Ms of four independent experiments. **** *p* < 0.0001 vs. untreated MeOV or MeTRAV cells. ◦◦◦◦ *p* < 0.0001 vs. respective treatments indicated by the bars.; ^^^ *p* < 0.001 vs. respective treatments indicated by the bars.

Considering that the cytotoxic effect of imidazo-pyrazoles **I** is mediated by ROS production [21], the amount of H_2_O_2_, the most long-lived ROS [25,26], was monitored in both cell lines under each treatment condition. As shown in Figure 4, **1h** exposure increased H_2_O_2_ levels in MeOV and MeTA cells in a time-dependent way, reaching, at 72 h, an increase of 3.5 fold and 4 fold, respectively.

#### 2.2.4. **1h** Is Not Cytotoxic for Healthy HaCAT Keratinocytes and Does Not Stimulate H_2_O_2_ Production in These Cells

**Figure 5:**
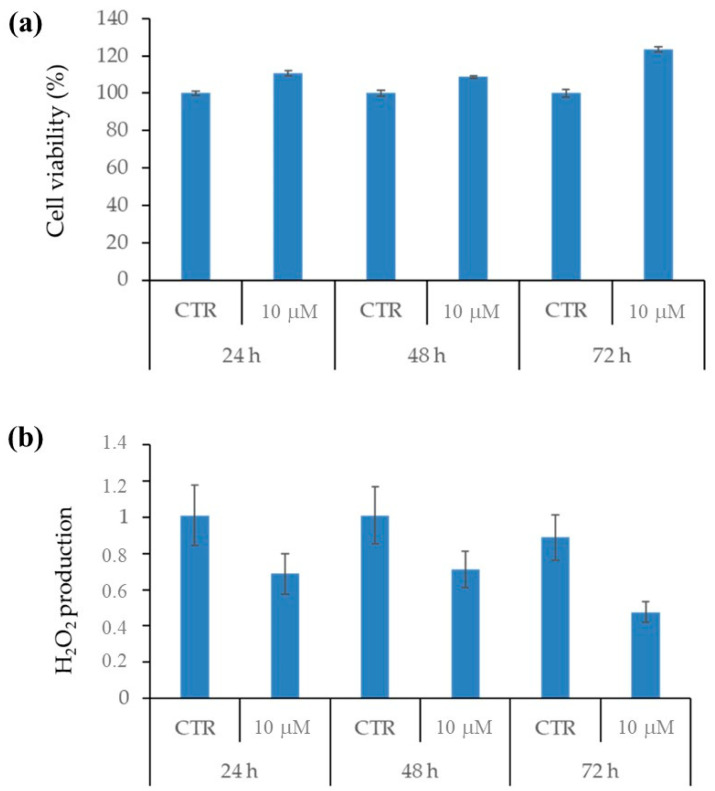
**1h** exposure did not alter viability and did not induce H_2_O_2_ production in healthy HaCAT keratinocytes. Cell viability (**a**) and H_2_O_2_ production (**b**) were evaluated by MTS assay and DCFH-DA labelling, respectively, in HaCAT cells treated with 10 µM **1h** for 24, 48, and 72 h. Histograms summarize quantitative data of means ± S.E.Ms of at least four independent experiments.

In order to evaluate the impact of **1h** on non-cancerous cells, the same treatment performed on melanoma cells was also performed on healthy HaCAT keratinocytes. As shown in Figure 5, **1h** treatment, at any time of exposure, did not affect HaCAT viability (Figure 5a) and did not induce significant changes in H_2_O_2_ production (Figure 5b).

### 2.3. In Silico Prediction of ADMET Properties of ***1h***

In silico predictions of absorption, distribution, metabolism, excretion, and toxicity (ADMET) properties are exploited as valuable tools in drug design [27,28,29]. To estimate the drug-like profile of compound **1h**, the ADMET properties of **1h** were calculated via the ACD/Lab Percepta platform [30]. Putative violations of Veber’s [31] and Lipinski’s rules [32] were considered and the following descriptors were calculated: the (i) logarithmic ratio of the octanol–water partitioning coefficient (cLogP), (ii) molecular weight (MW) values of the compounds, (iii) H-bonding acceptor number (HBA), H-bonding donor moieties (HBD), (iv) number of rotatable bonds (nRot_bond), and (v) topological polar surface area (TPSA) (see Supplementary Materials Table S2). In addition, further pharmacokinetic parameters were taken into account such as human intestinal absorption (HIA), the estimation of the plasmatic protein binding (% PPB), the volume of distribution (Vd), ligand affinity toward human serum albumin (LogKa HSA), and putative oral bioavailability, as a percentage (F %) (Supplementary Materials Table S2).

Regarding hepatic metabolism, the potential inhibitory activity of **1h** against certain isoenzymes of cytochrome P450, particularly CYP1A2, CYP2C19, CYP2C9, CYP2D6, and CYP3A4, was evaluated; it is important to emphasize that the compound was not predicted to inhibit CYP3A4, an enzyme of fundamental importance in the metabolism of numerous drugs, especially in patients undergoing polypharmacy.

In general, the comparison of the chemical–physical properties of **1h** with the thiomethyl derivatives **1a**–**g** evidenced that all these new triazolo-imidazo-pyrazoles here reported had similar profiles in terms of pharmacokinetic properties, metabolism, and druglikeness characteristics. However, it should be noted that **1h**, having an additional hydroxy group on the triazole nucleus, would exhibit better hydrophilicity and solubility (5.67 × 10^−3^ mg/mL), as predicted by the ESOL method [33,34], and a greater number of H-bond acceptors (seven for **1 h** instead of six for **1a**–**g**) and H-bond donors (two for **1h** instead of one for **1a**–**g**).

Finally, the in silico evaluation of toxicity properties in terms of cytochrome inhibition and of the lethal dose (LD_50_) via mouse oral administration was explored (Supplementary Materials Table S3).

### 2.4. Micellar Solubilization of ***1h***

**Figure 6:**
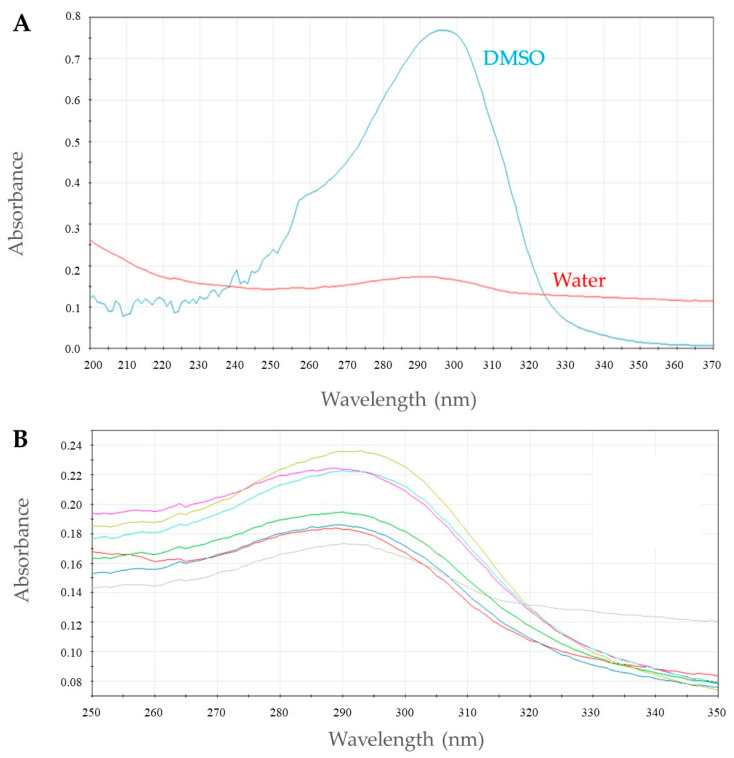
Plot of UV absorption spectra of **1h** in water and DMSO (**A**) and in micellar media of TW-80 at different concentrations. Each color represents a different concentration (**B**).

**Figure 7:**
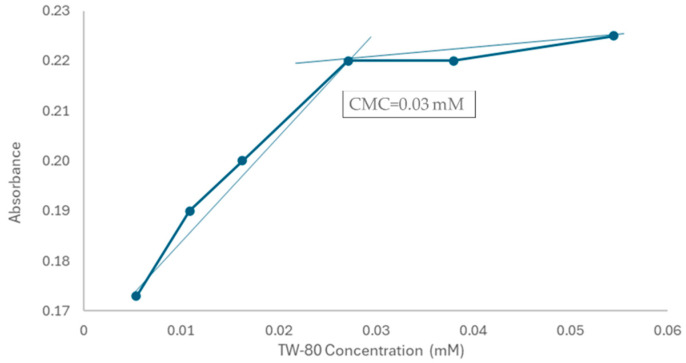
Plot of absorbance of **1h** against different concentrations of TW-80.

To evaluate the behavior of **1h** in solution, a pre-formulative study was carried out. Initially, the UV-visible spectra of **1h** in water and DMSO (0.05 mM) were recorded to identify the maximum absorbance value (λ max = 290 nm; Figure 6A). Then, the absorption profile of **1h** in the presence of different amount of the Tween 80 (TW-80) surfactant was measured (Figure 6B) and the critical micelle concentration (CMC) was determined (Figure 7). When the TW-80 concentration exceeds the CMC, a large number of micelles are formed, promoting compound encapsulation and enhancing its solubility and stability. As a result, this phenomenon leads to an increase in absorption efficiency [35].

## 3. Discussion

The development of a sequential synthetic approach based on key intermediate **2** allowed the isolation of a series of eight unreported imidazo-pyrazole derivatives characterized by a 1,2,4 triazole nucleus. The nature and the position of the *N*-phenyl ring substituent (X group, Scheme 1) affected the reactivity of the thiol group of compounds **4** and required the setup of two different protocols for the methylation reaction. In particular, unsubstituted (**4a**) and *ortho* or *para* fluoro-substituted (**4b** and **4d**) derivatives were successfully methylated in absolute ethanol using sodium acetate as a base. Conversely, compounds **4c**,**e**–**g** proved to be unreactive under these conditions and required the use of potassium carbonate as a base and dry DMF as a solvent. Interestingly, the nucleophilic substitution of the thiomethyl group of **1f** was observed by prolonging stirring in DMF for 24 h, leading to the isolation of the hydroxy derivative **1h**. Under the same experimental conditions, the thiomethyl displacement did not occur for the other derivatives, thus indicating the involvement of the 4-trifluoromethyl substituent in the reaction.

The substituents of the triazolyl substructure affected the anti-proliferative profile of the derivatives **1**). Thus, among the SMe series, compounds **1b** (X = 2-F), **1c** (X = 3-F), **1d** (X = 4-F), **1f** (X = 4-CF_3_), and **1g** (X = 4-Me) did not show any relevant anti-cancer activity against NCI cell line panel. Conversely, unsubstituted (**1a**) and 4-chlorosubstituted derivatives (**1e**) were found to affect the proliferation of selected cell lines (Table 1). The replacement of the SMe group with a hydroxyl functionality dramatically improved activity (compare **1f** and **1h**), leading to the identification of the most promising compound of the series. Derivative **1h** showed widespread anti-proliferative activity and proved to be lethal particularly for melanoma MDA-MB-435 cells.

The effectiveness of **1h** was confirmed in the human MeOV and MeTA melanoma cell lines, bearing different BRAF mutations. In fact, incubation with 10 µM **1h** markedly decreased the viability of both cell populations and this event was accompanied by a time-dependent increase in H_2_O_2_ levels. It is noteworthy that **1h** exerts its anti-proliferative and pro-oxidant activities selectively on melanoma cells while it is ineffective on healthy keratinocytes (HaCAT).

Computational studies (ACD/Lab Percepta platform) supported the drug-like profile of **1h** as no violations of drug-like property rules, such as Lipinski’s or Veber’s rules, were observed. Oral bioavailability was predicted to be 99%, with **1h** being chemically stable in acidic conditions and predicted with a dose/solubility ratio of 0.83. Passive absorption across the intestinal barrier was calculated to be greater than 70%. In silico toxicology results pointed out no cytochrome 3A4 and 2D6 inhibition events (based on the reported reliability indices) and an estimated LD_50_ value of 570 mg/Kg for mouse acute oral toxicity.

Poorly water-soluble drugs often require specialized formulation strategies to enhance bioavailability [36,37]. One promising approach involves the use of surfactants—i.e., amphiphilic compounds capable of reducing surface tension between two immiscible phases. These molecules contain both hydrophilic (water-attracting) and hydrophobic (water-repelling) regions within the same structure. Surfactants can improve drug solubility by forming colloidal-sized structures known as micelles in suitable solvents. When present at higher concentrations than the CMC, these amphiphilic molecules spontaneously self-assemble into micelles. Thanks to this micelle-forming ability, surfactants have recently gained significant attention as potential carriers in drug delivery systems [38,39]. On this basis, the behavior of the hydrophilic non-ionic surfactant TW-80 and its ability to form micelles and encapsulate **1h** were studied. In the presence of different TW-80 concentrations, the compound absorbance raised sharply in the pre-micellar region until reaching the CMC, after which it gradually decreased as the drug became fully incorporated into the micelles (Figure 6 and Figure 7). The typical CMC value of TW-80 in water is 0.015 mM [40], but after compound incorporation, the CMC increases at surfactant concentration values of 0.03 mM (Figure 7). It has been hypothesized that this could be due to the drug disrupting micelle formation, making the environment unfavorable for micellization, with the CMC consequently increasing [41]. Collectively, this preliminary study suggested a potential increase in **1h** solubility and will be helpful in selecting the most suitable drug delivery system for subsequent formulation studies.

## 4. Materials and Methods

### 4.1. General Information

All chemicals were purchased from Chiminord srl and Merk (formerly Aldrich Chemical) (Milan, Italy). Solvents were reagent-grade. All commercial reagents were used without further purification. Aluminum-backed silica gel plates (Merck DC-Alufolien Kieselgel 60 F254, Darmstad, Germany) were used in thin-layer chromatography (TLC) for routine of monitoring the reaction course. Merck silica gel, 230–400 mesh, was used for chromatography. Flash chromatography was performed using Isolera one instrument (Biotage, Uppsala, Sweden) using Silicagel column. Melting points were not “corrected” and were measured with a Buchi M-560 instrument (Buchi instruments, Flawil, Switzerland). ^1^H and ^13^C NMR spectra (Supplementary Materials Figures S1–S30) were recorded on a JEOL JNM ECZ-400S/L1 (400 MHz, Tokyo, Japan); chemical shifts are reported as δ (ppm) relative to tetramethylsilane (TMS) as internal standard; signals were characterized as s (singlet), d (doublet), t (triplet), n t (near triplet), q (quartet), m (multiplet), or br s (broad signal); J values were reported in Hz. IR spectra were recorded on Perkin-Elmer 398.HPLC/ESI-MS analyses were carried out with 1100 HPLC coupled with an Agilent 1100 series LC/MSD XCT Ion Trap (Agilent Technologies, Santa Clara, CA, USA).

Elemental analysis was determined with an elemental analyzer EA 1110 (Fison Instruments, Milan, Italy); products were considered pure when the difference between calculated and found values was ± 0.4 (see Supplementary Materials Table S1). Compound **2** was prepared as previously reported [20,42].

### 4.2. Chemistry

#### 4.2.1. General Procedure for Synthesis of Intermediates **3a**–**g**

To a solution of 2-phenyl-2,3-dihydro-1*H*-imidazo[1,2-*b*]pyrazole-7-carbohydrazide **2** (1.22 g, 5 mmol) in absolute ethanol (40 mL), the proper phenyl isothiocyanate (6 mmol) solved in absolute ethanol (2 mL) was added and the mixture was heated at reflux for 6–18 h. After cooling to room temperature, the obtained solid was filtered and recrystallized by absolute ethanol or by a 1/1 mixture of absolute ethanol/methanol.

*N-phenyl-2-(2-phenyl-2,3-dihydro-1H-imidazo[1,2-b]pyrazole-7-carbonyl)hydrazine-1-carbothioamide* **3a**. Yield: 92%. M.p.: 139–141 °C. ^1^H-NMR (400 MHz, DMSO-d_6_): δ 3.82 (t, *J* = 9.5 Hz, 1H, H_3_), 4.56 (t, *J* = 9.5 Hz, H, H_3_), 5.36–5.43 (m, 1H, H_2_), 7.06–7.12 (m, 1H, Ar), 7.19 (br s, 1H, NH exchangeable with D_2_O), 7.23–7.45 (m, 9H, Ar), 7.72 (s, 1H, H_6_), 9.51 (br s, 1H, NH exchangeable with D_2_O), 9.62 (br s, 1H, NH exchangeable with D_2_O), 9.68 (br s, 1H, NH exchangeable with D_2_O). ^13^C-NMR (101 MHz, DMSO-d_6_): δ 183.98, 164.62, 148.24, 146.82, 142.46, 140.91, 139.31, 130.38, 128.56, 127.86, 126.64, 125.63, 93.90, 64.95, 53.10. IR (KBr): 3430 (NH), 1652 (CO) cm^−1^. Anal calcd. for C_19_H_18_N_6_OS.

*N-(2-fluorophenyl)-2-(2-phenyl-2,3-dihydro-1H-imidazo[1,2-b]pyrazole-7-carbonyl)hydrazine-1-carbothioamide* **3b**. Yield: 66%. M.p.: 190–193 °C. ^1^H-NMR (400 MHz, DMSO-d_6_): δ 3.73 (t, *J* = 9.5 Hz, 1H, H_3_), 4.56 (t, *J* = 9.5 Hz, H, H_3_), 5.32–5.42 (m, 1H, H_2_), 7.09–7.41 (m, 10H, 9Ar + NH), 7.72 (s, 1H, H_6_), 9.49 (br s, 1H, NH exchangeable with D_2_O), 9.62–9.76 (m, 2H, 2xNH exchangeable with D_2_O). ^13^C-NMR (101 MHz, DMSO-d_6_): δ 183.00, 166.30, 159.56 (d, *J* = 247.9 Hz), 141.71, 140.92, 133.54, 130.42, 128.56, 127.86, 127.39, 126.63, 126.04, 123.84, 115.62 (d, *J* = 19.8 Hz), 93.81, 64.95, 53.10. IR (KBr): 3385 (NH), 1674 (CO) cm^−1^. Anal calcd. for C_19_H_17_FN_6_OS.

*N-(3-fluorophenyl)-2-(2-phenyl-2,3-dihydro-1H-imidazo[1,2-b]pyrazole-7-carbonyl)hydrazine-1-carbothioamide* **3c**. Yield: 73%. M.p.: 163–165 °C. ^1^H-NMR (400 MHz, DMSO-d_6_): δ 3.78 (t, *J* = 9.5 Hz, 1H, H_3_), 4.61 (t, *J* = 9.5 Hz, H, H_3_), 5.39–5.48 (m, 1H, H_2_), 6.97 (br s, 1H, NH exchangeable with D_2_O), 7.20–7.46 (m, 9H, Ar), 7.77 (s, 1H, H_6_), 9.63–9.84 (m, 3H, 3xNH exchangeable with D_2_O). ^13^C-NMR (101 MHz, DMSO-d_6_): δ 184.14, 166.27, 155.70, 142.16, 141.31, 140.90, 129.88, 129.14, 128.57, 127.87, 126.64, 121.09, 112.95, 103.58, 93.84, 64.96, 53.10.IR (KBr): 3356 (NH), 1655 (CO) cm^−1^. Anal calcd. for C_19_H_17_FN_6_OS.

*N-(4-fluorophenyl)-2-(2-phenyl-2,3-dihydro-1H-imidazo[1,2-b]pyrazole-7-carbonyl)hydrazine-1-carbothioamide* **3d**. Yield: 99%. M.p.: 150–153 °C. ^1^H-NMR (400 MHz, DMSO-d_6_): δ 3.77 (t, *J* = 9.5 Hz, 1H, H_3_), 4.59 (t, *J* = 9.5 Hz, H, H_3_), 5.38–5.48 (m, 1H, H_2_), 7.11–7.17 (m, 2H, Ar), 7.21 (br s, 1H, NH exchangeable with D_2_O), 7.30–7.47 (m, 7H, Ar), 7.75 (s, 1H, H_6_), 9.52–9.78 (m, 3H, 3xNH exchangeable with D_2_O). ^13^C-NMR (101 MHz, DMSO-d_6_): δ 182.12, 167.68, 157.91, 142.94, 141.45, 136.18, 133.12, 129.10, 128.41, 127.17, 120.63, 115.18, 94.43, 65.48, 53.64. IR (KBr): 3431 (NH), 1653 (CO) cm^−1^. Anal calcd. for C_19_H_17_FN_6_OS.

*N-(4-chlorophenyl)-2-(2-phenyl-2,3-dihydro-1H-imidazo[1,2-b]pyrazole-7-carbonyl)hydrazine-1-carbothioamide* **3e**. Yield: 63%. M.p.: 162–165 °C. ^1^H-NMR (400 MHz, DMSO-d_6_): δ 3.78 (t, *J* = 9.5 Hz, 1H, H_3_), 4.59 (t, *J* = 9.5 Hz, H, H_3_), 5.39–5.46 (m, 1H, H_2_), 7.22 (br s, 1H, NH exchangeable with D_2_O), 7.29–7.44 (m, 7H, Ar), 7.47–7.52 (m, 2H, Ar), 7.75 (s, 1H, H_6_), 9.61–9.70 (m, 2H, 2xNH exchangeable with D_2_O), 9.78 (br s, 1H, NH exchangeable with D_2_O). ^13^C-NMR (101 MHz, DMSO-d_6_): δ 184.10, 164.74, 155.92, 142.96, 141.44, 135.09, 129.10, 128.41, 128.30, 127.79, 127.17, 124.13, 94.47, 65.49, 53.64.IR (KBr): 3419 (NH), 1652 (CO) cm^−1^. Anal calcd. for C_19_H_17_ClN_6_OS.

*2-(2-phenyl-2,3-dihydro-1H-imidazo[1,2-b]pyrazole-7-carbonyl)-N-(4-(trifluoromethyl)phenyl)hydrazine-1-carbothioamide* **3f**. Yield: 55%. M.p.: 150–152 °C. ^1^H-NMR (400 MHz, DMSO-d_6_): δ 3.77 (t, *J* = 9.5 Hz, 1H, H_3_), 4.59 (t, *J* = 9.5 Hz, H, H_3_), 5.37–5.47 (m, 1H, H_2_), 7.10–7.18 (m, 2H, Ar), 7.22 (br s, 1H, NH exchangeable with D_2_O), 7.28–7.48 (m, 7H, Ar), 7.75 (s, 1H, H_6_), 9.59 (br s, 1H, NH exchangeable with D_2_O), 9.67 (m, 1H, NH exchangeable with D_2_O), 9.73 (br s, 1H, NH exchangeable with D_2_O). ^13^C-NMR (101 MHz, DMSO-d_6_): δ 182.21, 160.62, 142.93, 141.44, 136.19, 129.62, 129.10, 128.41, 127.17, 126.18, 121.22, 114.98, 94.43, 65.48, 53.63. IR (KBr): 3335 (NH), 1657 (CO) cm^−1^. Anal calcd. for C_20_H_17_F_3_N_6_OS.

*2-(2-phenyl-2,3-dihydro-1H-imidazo[1,2-b]pyrazole-7-carbonyl)-N-(p-tolyl)hydrazine-1-carbothioamide* **3g**. Yield: 73%. M.p.: 152–154 °C. ^1^H-NMR (400 MHz, DMSO-d_6_): δ 2.24 (s, 3H, CH_3_), 3.77 (t, *J* = 9.5 Hz, 1H, H_3_), 4.59 (t, *J* = 9.5 Hz, 1H, H_3_), 5.37–5.42 (m, 1H, H_2_), 7.07–7.41 (m, 9H Ar), 7.72 (s, 1H, H_6_), 9.45 (br s, 1H, NH exchangeable with D_2_O), 9.61 (br s, 1H, NH exchangeable with D_2_O). ^13^C-NMR (101 MHz, DMSO-d_6_): δ 182.26, 161,88, 154.55, 142.43, 140.93, 133.95, 136.74, 128.59, 128.39, 127.89, 126.66, 125.82, 93.94, 64.96, 53.12, 20.57. Anal calcd. for C_21_H_20_N_6_OS.

#### 4.2.2. General Procedure for Synthesis of Intermediates **4a**–**g**

The proper compound **3a–g** (2 mmol) was suspended in 2M NaOH (16 mL) and the mixture was heated at 80 °C for 4–12 h (TLC monitoring). After cooling to room temperature, concentrated AcOH was added until a pH of 5 was reached. The obtained solid was filtered and crystallized by absolute ethanol or by a 1/1 mixture of absolute ethanol/methanol.

*4-phenyl-5-(2-phenyl-2,3-dihydro-1H-imidazo[1,2-b]pyrazol-7-yl)-2,4-dihydro-3H-1,2,4-triazole-3-thione* **4a.** Yield: 92%. M.p.: 264–267 °C. ^1^H-NMR (400 MHz, DMSO-d_6_): δ 3.71–3.76 (m, 1H, H_3_), 4.55–4.60 (m, 1H, H_3_), 5.38–5.43 (m, 1H, H_2_), 6.16 (br s, 1H, NH exchangeable), 6.90 (br s, 1H, NH exchangeable), 7.31–7.44 (m, 8H, 7Ar + H_6_), 7.59–7.63 (m, 3H, Ar). ^13^C-NMR (101 MHz, DMSO-d_6_): δ 167.09, 152.82, 146.13, 140.81, 139.42, 134.72, 129.88, 129.67, 128.97, 128.51, 127.83, 126.66, 85.64, 64.92, 53.23. IR (KBr): 3278 (NH) cm^−1^. Anal calcd. for C_19_H_16_N_6_S.

*4-(2-fluorophenyl)-5-(2-phenyl-2,3-dihydro-1H-imidazo[1,2-b]pyrazol-7-yl)-2,4-dihydro-3H-1,2,4-triazole-3-thione* **4b**. Yield: 55%. M.p.: 266–272 °C. ^1^H-NMR (400 MHz, DMSO-d_6_): δ 3.73–3.80 (m, 1H, H_3_), 4.57–4.62 (m, 1H, H_3_), 5.41–5.45 (m, 1H, H_2_), 6.22 (br s, 1H, NH exchangeable), 6.27 (br s, 1H, NH exchangeable), 7.03–7.07 (m, 1H, Ar), 7.31–7.72 (m, 9H, 9Ar + H_6_). ^13^C-NMR (101 MHz, DMSO-d_6_): δ 167.39, 158.71, 152.91, 146.11, 140.75, 138.60, 132.70, 131.66, 128.53, 127.86, 126.71, 125.65, 122.30, 116.98, 85.30, 65.03, 53.25. IR (KBr): 3282 (NH) cm^−1^. Anal calcd. for C_19_H_15_FN_6_S.

*4-(3-fluorophenyl)-5-(2-phenyl-2,3-dihydro-1H-imidazo[1,2-b]pyrazol-7-yl)-2,4-dihydro-3H-1,2,4-triazole-3-thione* **4c**. Yield: 66%. M.p.: 273–274 °C. ^1^H-NMR (400 MHz, DMSO-d_6_): δ 3.71–3.76 (m, 1H, H_3_), 4.56–4.61 (m, 1H, H_3_), 5.38–5.43 (m, 1H, H_2_), 6.34 (br s, 1H, NH exchangeable), 6.90 (br s, 1H, NH exchangeable), 7.26–7.49 (m, 9H, 8Ar + H_6_), 7.61–7.67 (m, 1H, Ar). ^13^C-NMR (101 MHz, DMSO-d_6_): δ 167.05, 163.35, 152.76, 145.89, 140.87, 139.62, 136.25, 131.27, 128.52, 127.84, 126.63, 125.45, 125.41, 116.80, 85.56, 64.89, 53.29. IR (KBr): 3287 (NH) cm^−1^. Anal calcd. for C_19_H_15_FN_6_S.

*4-(4-fluorophenyl)-5-(2-phenyl-2,3-dihydro-1H-imidazo[1,2-b]pyrazol-7-yl)-2,4-dihydro-3H-1,2,4-triazole-3-thione* **4d**. Yield: 66%. M.p.: 294–295 °C. ^1^H-NMR (400 MHz, DMSO-d_6_): δ 3.68–3.77 (m, 1H, H_3_), 4.54–4.63 (m, 1H, H_3_), 5.35–5.41 (m, 1H, H_2_), 6.33 (br s, 1H, NH exchangeable), 6.71 (br s, 1H, NH exchangeable), 7.11–7.48 (m, 10H, 9Ar + H_6_).^13^C-NMR (101 MHz, DMSO-d_6_): δ 173.93, 166.86, 163.17, 152.31, 145.63, 141.06, 139.47, 131.25, 128.50, 127.78, 126.63, 116.20, 87.11, 64.81, 53.29. IR (KBr): 3268 (NH) cm^−1^. Anal calcd. for C_19_H_15_FN_6_S.

*4-(4-chlorophenyl)-5-(2-phenyl-2,3-dihydro-1H-imidazo[1,2-b]pyrazol-7-yl)-2,4-dihydro-3H-1,2,4-triazole-3-thione* **4e**. Yield: 60%. M.p.: 287–291 °C. ^1^H-NMR (400 MHz, DMSO-d_6_): δ 3.69–3.74 (m, 1H, H_3_), 4.56–4.61 (m, 1H, H_3_), 5.37–5.42 (m, 1H, H_2_), 6.45 (br s, 1H, NH exchangeable), 6.84 (br s, 1H, NH exchangeable), 7.30–7.53 (m, 8H, 7Ar + H_6_), 7.65–7.70 (m, 2H, Ar). ^13^C-NMR (101 MHz, DMSO-d_6_): δ 167.12, 152.72, 146.01, 140.83, 139.91, 134.52, 133.50, 130.93, 129.68, 128.55, 127.85, 126.61, 85.39, 64.89, 53.32. IR (KBr): 3344 (NH) cm^−1^. Anal calcd. for C_19_H_15_ClN_6_S.

*5-(2-phenyl-2,3-dihydro-1H-imidazo[1,2-b]pyrazol-7-yl)-4-(4-(trifluoromethyl)phenyl)-2,4-dihydro-3H-1,2,4-triazole-3-one* **4f**. Yield: 63%. M.p.: 163–166 °C. ^1^H-NMR (400 MHz, DMSO-d_6_): δ 3.79–3.73 (m, 1H, H_3_), 4.49–4.52 (m, 1H, H_3_), 5.48–5.51 (m, 1H, H_2_), 6.53 (br s, 1H, NH exchangeable), 7.23–7.37 (m, 8H, 7Ar + H_6_), 7.61–7.64 (m, 2H, Ar). ^13^C-NMR (101 MHz, DMSO-d_6_): δ 166.36, 155.18, 138.87, 135.58, 133.61, 128.43, 128.34, 127.77, 126.44, 125.05, 123.62, 122.97, 119.62, 86.24, 67.25, 58.03. IR (KBr): 3381 (NH) cm^−1^. Anal calcd. for C_20_H_15_F_3_N_6_.

*5-(2-phenyl-2,3-dihydro-1H-imidazo[1,2-b]pyrazol-7-yl)-4-(p-tolyl)-2,4-dihydro-3H-1,2,4-triazole-3-thione* **4g**. Yield: 52%. M.p.: 247–249 °C. ^1^H-NMR (400 MHz, DMSO-d_6_): δ 2.42 (s, 3H, CH_3_), 3.70–3.75 (m, 1H, H_3_), 4.57–4.59 (m, 1H, H_3_), 5.37–5.42 (m, 1H, H_2_), 6.25 (br s, 1H, NH exchangeable), 6.81 (br s, 1H, NH exchangeable), 7.25–7.40 (m, 11H, 9Ar + H_6_). ^13^C-NMR (101 MHz, DMSO-d_6_): δ 167.67, 153.23, 146.71, 141.37, 140.10, 132.85, 130.61, 129.19, 129.05, 128.36, 127.18, 125.70, 86.49, 65.46, 53.78, 21.39. Anal calcd. for C_20_H_18_N_6_S.

#### 4.2.3. Synthesis of Compounds **1a**,**b**,**d**

To an absolute ethanol solution (10 mL) of the proper compounds **4** (1 mmol), sodium acetate (0.500 g, 6 mmol) and iodomethane (0.150 g, 1 mmol) were added and the mixture was heated at reflux for 3–4 h, monitoring, by TLC, the product formation. After cooling to room temperature, the solvent was evaporated under reduced pressure and the crude was solved in dichloromethane (20 mL); the organic phase was washed with water (2 × 10 mL), then dried (MgSO_4_) and concentrated under reduced pressure. The obtained solid was filtered and recrystallized by absolute ethanol or diethyl ether.

*7-(5-(methylthio)-4-phenyl-4H-1,2,4-triazole-3-yl)-2-phenyl-2,3-dihydro-1H-imidazo[1,2-b]pyrazole* **1a**. Yield: 53%. M.p.: 98–100 °C. ^1^H-NMR (400 MHz, CDCl_3_): δ 2.69 (s, 3H, SCH_3_), 3.92–3.97 (m, 1H, H_3_), 4.55–4.60 (m, 1H, H_3_), 5.66–5.70 (m, 1H, H_2_), 6.34 (br s, 1H, NH exchangeable), 7.32–7.48 (m, 8H, 7Ar + H_6_), 7.68–7.75 (m, 3H, Ar). ^13^C-NMR (101 MHz, CDCl_3_): δ 154.72, 154.11, 147.41, 140.46, 139.32, 132.34, 131.65, 131.23, 129.09, 128.63, 127.60, 126.60, 125.48, 65.96, 54.23, 14.48. IR (KBr): 3213 (NH) cm^−1^. Anal calcd. for C_20_H_18_N_6_S.

*7-(4-(2-fluorophenyl)-5-(methylthio)-4H-1,2,4-triazole-3-yl)-2-phenyl-2,3-dihydro-1H-imidazo[1,2-b]pyrazole* **1b**. Yield: 52%. M.p.: 195–196 °C. ^1^H-NMR (400 MHz, CDCl_3_): δ 2.53 (s, 3H, SCH_3_), 3.80–3.85 (m, 1H, H_3_), 4.40–4.45 (m, 1H, H_3_), 5.42–5.47 (m, 1H, H_2_), 6.32 (br s, 1H, NH exchangeable), 7.19–7.34 (m, 9H, 8Ar + H_6_), 7.51–7.54 (m, 1H, Ar). ^13^C-NMR (101 MHz, CDCl_3_): δ 157.84 (d, *J* = 255.8 Hz), 153.86, 152.64, 149.28, 139.43 (m), 133.69 (m), 129.79 (d, *J* = 7.2 Hz), 129.40, 129.11, 128.71, 126.58, 126.00 (m), 125.47, 117.98 (m), 104.83, 66.14, 54.37, 14.94. IR (KBr): 3326 (NH) cm^−1^. Anal calcd. for C_20_H_17_FN_6_S.

*7-(4-(4-fluorophenyl)-5-(methylthio)-4H-1,2,4-triazole-3-yl)-2-phenyl-2,3-dihydro-1H-imidazo[1,2-b]pyrazole* **1d**. Yield: 70%. M.p.: 195–197 °C. ^1^H-NMR (400 MHz, DMSO-d_6_): δ 2.61 (s, 3H, SCH_3_), 3.99–4.03 (m, 1H, H_3_), 4.61–4.65 (m, 1H, H_3_), 5.62–5.66 (m, 1H, H_2_), 6.50 (br s, 1H, NH exchangeable), 7.24–7.37 (m, 4H, Ar), 7.45–7.52 (m, 3H, 2Ar + H_6_), 7.56–7.59 (m, 3H, Ar). ^13^C-NMR (101 MHz, DMSO-d_6_): δ 161.56 (d, *J* = 246.5 Hz), 147.93, 147.10, 138.47, 138.23, 138.11, 133.76 (d, *J* = 3.0 Hz), 129.34 (d, *J* = 8.4 Hz), 127.50, 126.35, 126.01, 115.92 (d, *J* = 22.5 Hz), 89.62, 64.10, 56.53, 20.29. IR (KBr): 3361 (NH) cm^−1^. Anal calcd. for C_20_H_17_FN_6_S.

#### 4.2.4. Synthesis of Compounds **1c**,**e**–**g**

To an anhydrous DMF solution (2 mL) of the proper compound **4** (1 mmol), anhydrous potassium carbonate (0.171 g, 1.24 mmol) and iodomethane (0.300 g, 2.13 mmol) were added and the mixture was stirred at rt for 1 h (compounds **1c** and **1f**) or 18 h (compound **1e** and **1g**), monitoring, by TLC, the product. Then water was added (20 mL); the precipitated solid was filtered, washed 3 times with water, and recrystallized from absolute ethanol.

*7-(4-(3-fluorophenyl)-5-(methylthio)-4H-1,2,4-triazole-3-yl)-2-phenyl-2,3-dihydro-1H-imidazo[1,2-b]pyrazole* **1c**. Yield: 62%. M.p.: 105–108 °C. ^1^H-NMR (400 MHz, CDCl_3_): δ 2.67 (s, 3H, SCH_3_), 3.95–4.00 (m, 1H, H_3_), 4.54–4.59 (m, 1H, H_3_), 5.53–5.57 (m, 1H, H_2_), 6.49 (br s, 1H, NH exchangeable), 7.12–7.14 (m, 1H, Ar), 7.32–7.43 (m, 7H, 6Ar + H_6_), 7.58–7.60 (m, 1H, Ar). ^13^C-NMR (101 MHz, CDCl_3_): δ 163.16 (d, *J* = 251.8 Hz), 153.54, 151.81, 149.58, 139.57, 139.59, 134.49 (d, *J* = 9.1 Hz), 131.92 (d, *J* = 8.7 Hz), 129.12, 128.74, 126.56, 123.76, 118.42 (d, *J* = 20.7 Hz), 115.56 (*J* = 23.7 Hz), 85.87, 66.17, 54.83, 14.87. IR (KBr): 3267 (NH) cm^−1^. Anal calcd. for C_20_H_17_FN_6_S.

*7-(4-(4-chlorophenyl)-4H-1,2,4-triazole-3-yl)-2-phenyl-2,3-dihydro-1H-imidazo[1,2-b]pyrazole* **1e**. Yield: 63%. M.p.: 127–129 °C. ^1^H-NMR (400 MHz, CDCl_3_): δ 2.69 (s, 3H, SCH_3_), 3.94–3.98 (m, 1H, H_3_), 4.55–4.60 (m, 1H, H_3_), 5.62–5.65 (m, 1H, H_2_), 6.46 (br s, 1H, NH exchangeable), 7.32–7.48 (m, 7H, 6Ar + H_6_), 7.62–7.72 (m, 3H, Ar). ^13^C-NMR (101 MHz, CDCl_3_): δ 154.24, 153.19, 148.31, 140.14, 139.37, 138.22, 131.91, 131.30, 130.67, 129.12, 129.04, 128.70, 126.58, 66.06, 54.29, 14.68. IR (KBr): 3342 (NH) cm^−1^. Anal calcd. for C_20_H_17_ClN_6_S.

*7-(5-(methylthio)-4-(4-(trifluoromethyl)phenyl)-4H-1,2,4-triazole-3-yl)-2-phenyl-2,3-dihydro-1H-imidazo[1,2-b]pyrazole* **1f**. Yield: 42%. M.p.: 147–149 °C. ^1^H-NMR (400 MHz, CDCl_3_): δ 2.67 (s, 3H, SCH_3_), 3.71–3.75 (m, 1H, H_3_), 4.40–4.44 (m, 1H, H_3_), 5.19–5.22 (m, 1H, H_2_), 6.47 (br s, 1H, NH exchangeable), 7.24–7.37 (m, 8H, 7Ar + H_6_), 7.81–7.85 (m, 2H, Ar). ^13^C-NMR (101 MHz, CDCl_3_): δ 153.65, 151.92, 149.44, 139.91, 139.59, 136.34, 129.33, 129.12 (d, *J* = 5.7 Hz), 128.75 (d, *J* = 4.3 Hz), 128.50, 127.82, 126.63 (d, *J* = 6.7 Hz), 119.84 (d, *J* = 169.5 Hz), 114.41, 66.19, 54.36, 14.93. Anal calcd. for C_21_H_17_F_3_N_6_S.

*7-(5-(methylthio)-4-(p-tolyl)-4H-1,2,4-triazole-3-yl)-2-phenyl-2,3-dihydro-1H-imidazo[1,2-b]pyrazole* **1g**. Yield: 52%. M.p.: 87–89 °C. ^1^H-NMR (400 MHz, CDCl_3_): δ 2.43 (s, 3H, CH_3_), 2.54 (s, 3H, CH_3_), 3.70–3.75 (m, 1H, H_3_), 4.56–4.61 (m, 1H, H_3_), 5.38–5.43 (m, 1H, H_2_), 6.40 (br s, 1H, NH exchangeable), 7.30–7.43 (m, 10H, 9Ar + H_6_). ^13^C-NMR (101 MHz, DMSO-d_6_): δ 153.90, 150.44, 141.55, 140.66, 131.74, 131.06, 129.95, 129.03, 128.21, 127.30, 125.72, 117.30, 86.98, 65.68, 53.83, 21.38, 15.31. Anal calcd. for C_21_H_20_N_6_S.

#### 4.2.5. Synthesis of 5-(2-Phenyl-2,3-dihydro-1H-imidazo[1,2-b]pyrazol-7-yl)-4-(4-(trifluoromethyl)phenyl)-4H-1,2,4-triazole-3-ol **1h**

To an anhydrous DMF (2 mL) solution of compound **4f** (428 mg, 1 mmol), anhydrous potassium carbonate (0.171 g, 1.24 mmol) and iodomethane (300 mg, 2.13 mmol) were added and the mixture was stirred at rt for 24 h, monitoring, by TLC, the product. Then water was added (20 mL); a pale-rose solid was precipitated and filtered, washed 3 times with water, and crystallized from absolute ethanol.

Yield: 55%. M.p.: 223–225 °C. ^1^H-NMR (400 MHz, DMSO-d_6_): δ 3.86–3.91 (m, 1H, H_3_), 4.68–4.73 (m, 1H, H_3_), 5.48–5.53 (m, 1H, H_2_), 7.32–7.49 (m, 6H, 5Ar + NH exchangeable), 7.66–7.78 (m, 5H, 4Ar + H_6_), 10.92 (br s, 1H, NH exchangeable). ^13^C-NMR (101 MHz, DMSO-d_6_): δ 157.63, 154.02, 152.43, 142.48, 140.73, 140.69, 128.59, 127.94, 126.75, 126.59, 126.41, 121.23 (d, *J* = 207.9 Hz), 116.68, 84.27, 65.17, 53.39. IR (KBr): 3450 (NH) cm^−1^. Anal calcd. for C_20_H_15_F_3_N_6_O. HPLC/ESI-MS analysis [M + H]^+^ 413.3.

### 4.3. Biological Evaluation

#### 4.3.1. NCI Anti-Proliferative Screening

All compounds, **1a**–**h**, were tested for anti-proliferative activity by National Cancer Institute (Developmental Therapeutics Program, Division of Cancer Treatment and Diagnosis) at a fixed concentration of 10 µM [24].

#### 4.3.2. Cell Culture Conditions

MeOV and MeTA cells were kindly provided by Prof. Gabriella Pietra (University of Genoa, Genoa, Italy). HaCAT keratinocytes were kindly provided by Dr. Marco Ponassi (Biologic Bank and Cell Factory, IRCCS Policlinico San Martino, Genoa, Italy). Cell lines were maintained in RPMI 1640 medium (Euroclone Spa, Pavia, Italy) supplemented with 10% Fetal Bovine Serum (FBS, Euroclone Spa, Pavia, Italy), 1% L-Glutamine (Euroclone Spa, Pavia, Italy), and 1% Penicillin/Streptomicin (Euroclone Spa, Pavia, Italy) and grown in standard conditions (37 °C humidified incubator with 5% CO_2_).

#### 4.3.3. Treatments

MEOV and MeTA cells were treated for 24, 48, and 72 h with increasing concentrations (0–100 μM) of **1h**. Cell cultures were carefully monitored before and during the experiments to ensure optimal cell density. Notably, samples were discarded if the cell confluence reached >90%.

#### 4.3.4. Cell Viability Assay

Cell viability was determined by using the CellTiter 96^®^ AQueous One Solution Cell Proliferation Assay (Promega, Madison, WI, USA) and following the supplier’s instructions. Briefly, cells (10,000 cells/well) were seeded into 96-well plates (Corning Incorporated, Corning, NY, USA) and then treated. Next, cells were incubated with 20 mL of CellTiter and the absorbance at 490 nm was recorded using a microplate reader (EL-808, BIO-TEK Instruments Inc., Winooski, VT, USA).

#### 4.3.5. Detection of Hydrogen Peroxide (H_2_O_2_) Production

The production of H_2_O_2_ was evaluated using 2′-7′-dichlorofluorescein-diacetate (DCFH-DA; Merk Life Science S.r.l., Milan, Italy) as previously reported [43].

#### 4.3.6. Statistical Analysis

The results are expressed as means ± S.E.Ms of at least four independent experiments. The statistical significance of the parametric differences between the experimental data sets was assessed by one-way ANOVA followed by Dunnett’s test for multiple comparisons using GraphPad Prism 8.0.1 (GraphPad Software v8.0, San Diego, CA, USA). *p* < 0.05 was considered statistically significant.

### 4.4. In Silico Prediction of ADMET Properties

The prediction of ADMET parameters was carried out by means of the Advanced Chemistry Development (ACD) Percepta platform [30]. The software prediction is based on the software implemented training libraries, which include experimentally determined pharmacokinetic and safety properties for different series of compounds.

### 4.5. Preparation of Micellar Solutions

The stock solution of **1h**, at the concentration of 0.05 mM, was prepared for spectroscopic measurement. The surfactant solutions were prepared in deionized water and diluted with compound’s solution. The TW-80 concentrations were set to range from 0.014 to 0.14 mM. The solutions were kept for 2 h before being used to take measurements. The UV absorption spectra were carried out using a Thermo Scientific UV/VIS spectrophotometer (Evolution 300, Fischer Scientific, GmbH, Schwerte, Germany) at λ = 290 nm.

## 5. Conclusions

The SAR extension study on imidazo-pyrazole compounds led to the identification of the novel derivative **1h**, characterized by a substituted 1,2,4-triazolyl substructure. The compound showed selective anti-proliferative activity against different melanoma cell lines without affecting the viability of healthy keratinocytes. Furthermore, the **1h** increased the H_2_O_2_ levels in BRAF-mutated MEOV and MeTA cells.

As indicated by in silico predictions, **1h** would be endowed with promising ADME and toxicological profiles. Furthermore, the preliminary solubility of **1h** Tween-80 has provided a solid foundation for the rational selection of the most suitable drug delivery system in subsequent formulation studies.

Overall, the collected data support the pharmacological value of derivative **1h** as a novel lead compound for the development of new and effective anti-melanoma agents.

## Figures and Tables

**Table 1:** Cell growth percent values of most active derivatives on different cancer cell lines at 10 μM concentration for 72 h. For each compound, only cell lines with a growth percent values ≤60% are indicated. * A negative mean value indicates lethality.

	Growth Percent (%)		
*Tumor*/Cell Line	1a	1e	1h
*Leukaemia*			
K-562	>60	>60	3.03
CCFR-CEM	18.23	>60	47.96
MOLT-4	>60	>60	41.87
RPMI-8226	>60	>60	56.88
SR	>60	>60	17.22
*Non-small-cell lung cancer*			
A549/ATCC	>60	>60	45.49
EKVX	>60	>60	55.99
NCI-H522	55.05	>60	26.08
*Colon Cancer*			
HCT-116	>60	>60	46.00
HCT-15	>60	>60	35.08
HT29	>60	>60	28.39
KM12	>60	>60	49.25
SW-620	>60	>60	50.88
*CNS cancer*			
SF-295	>60	>60	32.01
U251	>60	>60	58.04
*Melanoma*			
MDA-MB-435 *	>60	>60	−25.66
SKMEL-2	>60	>60	50.21
SKMEL-5	>60	>60	31.31
UACC-257	>60	>60	51.37
UACC-62	>60	63.33	40.27
*Ovarian cancer*			
OVCAR-3	>60	>60	37.58
OVCAR-4	>60	>60	59.09
NCI/ADR-RES	>60	>60	56.01
*Renal cancer*			
A498	>60	55.24	46.80
ACHN	>60	>60	52.27
CAKI-1	>60	>60	32.04
UO-31	>60	>60	53.70
*Prostate cancer*			
PC-3	>60	>60	46.28
*Breast cancer*			
MCF7	>60	>60	26.87
T-47D	>60	>60	54.23

## Data Availability

Data are contained within the article and Supplementary Materials.

## Supplementary Materials

The following supporting information can be downloaded at: https://www.mdpi.com/article/10.3390/ijms26136312/s1.
