# Supplementary material for: Triazole-imidazo[1,2-b]pyrazoles Able to Counteract Melanoma Cell Survival Without Compromising the Viability of Healthy Keratinocytes
Source: Int J Mol Sci. 2025 Jun 30;26(13):6312. doi: 10.3390/ijms26136312 (PMC12249857; doi:10.3390/ijms26136312)

Supporting Materials

Triazol-imidazo[1,2-*b*]pyrazoles able to counteract melanoma cells survival without compromising the viability of healthy keratinocytes.

Chiara Brullo *, Barbara Marengo, Cinzia Domenicotti, Matteo Lusardi, Elena Cichero, Annalisa Salis, Debora Caviglia, Eleonora Russo and Andrea Spallarossa

Table S1. Elemental analysis of compounds **1, 3** and **4**
Table S2. Calculated properties based on the Lipinski's and Veber's rules of **1h**
Table S3. Calculated **1h** ADMET descriptors related to cytochrome inhibition or substrate ability and LD50 prediction
Figure S1: ^{1}H NMR (400 MHz) of compound **1a**
Figure S2: ^{13}C NMR (101 MHz) of compound **1a**
Figure S3: ^{1}H NMR (400 MHz) of compound **1b**
Figure S4: ^{13}C NMR (101 MHz) of compound **1b**
Figure S5: ^{1}H NMR (400 MHz) of compound **1c**
Figure S6: ^{13}C NMR (101 MHz) of compound **1c**
Figure S7: ^{1}H NMR (400 MHz) of compound **1d**
Figure S8: ^{13}C NMR (101 MHz) of compound **1d**
Figure S9: ^{1}H NMR (400 MHz) of compound **1e**
Figure S10: ^{13}C NMR (101 MHz) of compound **1e**
Figure S11: ^{1}H NMR (400 MHz) of compound **1f**
Figure S12: ^{13}C NMR (101 MHz) of compound **1f**
Figure S13: ^{1}H NMR (400 MHz) of compound **1g**
Figure S14: ^{13}C NMR (101 MHz) of compound **1g**
Figure S15: ^{1}H NMR (400 MHz) of compound **1h**
Figure S16: ^{13}C NMR (101 MHz) of compound **1h**
Figure S17: ^{1}H NMR (400 MHz) of compound **4a**
Figure S18: ^{13}C NMR (101 MHz) of compound **4a**
Figure S19: ^{1}H NMR (400 MHz) of compound **4b**
Figure S20: ^{13}C NMR (101 MHz) of compound **4b**
Figure S21: ^{1}H NMR (400 MHz) of compound **4c**
Figure S22: ^{13}C NMR (101 MHz) of compound **4c**
Figure S23: ^{1}H NMR (400 MHz) of compound **4d**
Figure S24: ^{13}C NMR (100 MHz) of compound **4d**
Figure S25: ^{1}H NMR (400 MHz) of compound **4e**
Figure S26: ^{13}C NMR (101 MHz) of compound **4e**
Figure S27: ^{1}H NMR (400 MHz) of compound **4f**
Figure S28: ^{13}C NMR (101 MHz) of compound **4f**
Figure S29: ^{1}H NMR (400 MHz) of compound **4g**
Figure S30: ^{13}C NMR (101 MHz) of compound **4g**
Figure S31: mean graphs of tested of **1a** in one-dose assay
Figure S32: mean graphs of tested of **1e** in one-dose assay
Figure S33: mean graphs of tested of **1h** in one-dose assay

Table S1. Elemental analysis of compounds **1, 3** and **4.**

Comp.	Values	%C	%H	%N	%S
**1a**	Calcd.	64.15	4.85	22.44	8.56
	Found	64.00	4.55	22.71	8.60
**1b**	Calcd.	61.21	4.37	21.41	8.17
	Found	61.18	4.60	21.11	7.93
**1c**	Calcd.	61.21	4.37	21.41	8.17
	Found	61.02	4.42	21.60	8.40
**1d**	Calcd.	61.21	4.37	21.41	8.17
	Found	61.31	4.40	21.58	8.21
**1e**	Calcd.	58.75	4.19	20.55	7.84
	Found	58.59	4.27	20.84	7.70
**1f**	Calcd.	57.01	3.87	18.99	7.25
	Found	57.00	3.50	18.77	7.00
**1g**	Calcd.	64.93	5.19	21.63	8.25
	Found	64.47	5.00	21.66	8.30
**1h**	Calcd.	58.25	3.67	20.38	//
	Found	58.55	3.25	20.11	//
**4a**	Calcd.	63.31	4.47	23.32	8.89
	Found	63.03	4.83	23.28	8.77
**4b**	Calcd.	60.30	4.00	22.21	8.47
	Found	60.15	4.15	22.16	8.36
**4c**	Calcd.	60.30	4.00	22.21	8.47
	Found	60.34	4.01	22.29	8.70
**4d**	Calcd.	60.30	4.00	22.21	8.47
	Found	60.26	3.70	22.40	8.42
**4e**	Calcd.	57.79	3.83	21.28	8.12
	Found	57.79	3.90	20.98	8.04
**4f**	Calcd.	56.07	3.53	19.62	7.48
	Found	56.20	3.63	20.00	7.12
**4g**	Calcd.	64.15	4.85	22.44	8.56
	Found	64.48	4.47	22.25	8.17
**3a**	Calcd.	60.30	22.21	4.79	8.47
	Found	60.61	22.41	5.05	8.56
**3b**	Calcd.	57.56	4.32	21.20	8.09
	Found	57.23	4.36	21.49	7.90
**3c**	Calcd.	57.56	4.32	21.20	8.09
	Found	57.71	4.38	21.10	8.22
**3d**	Calcd.	57.56	4.32	21.20	8.09
	Found	57.99	4.52	20.92	7.80
**3e**	Calcd.	55.27	4.15	20.35	7.76
	Found	55.37	4.23	20.33	7.70
**3f**	Calcd.	53.81	3.84	18.82	7.18
	Found	53.84	4.15	19.14	8.86
**3g**	Calcd.	61.21	5.14	21.41	8.17
	Found	61.07	4.85	21.30	8.05

Table S2. Calculated properties based on the Lipinski's and Veber's rules of **1h**. Reliability index values for a number of descriptors are shown as R.I. (values higher than 0.30 are ranked as reliable by the software).

**Comp.**	**MW**[a]	**N. H-bond acceptor**[b]	**N. H-bond donor**[c]	**N. rotable bonds**[d]	**cLogP GALAS**[e] (R.I. = 0.52)	**TPSA**[f]	**HIA (%)**[g]	**Vd (l/kg)**[h]	**%PPB**[i] (R.I. = 0.34)	**LogKa**[j] **HAS** (R.I. = 0.34)	**%F (oral)**[k] **50mg**
**1h**	411.38	6	2	4	3.41	67.38	100	2.7	98.50	5.07	99.1

[a] Molecular weight; [b] Number of H-bond acceptors; [c] Number of H-bond donors; [d] Number of rotatable bonds; [e] Logarithmic ratio of the octanol–water partitioning coefficient; [f] Topological polar surface area; [g] HIA represents the human intestinal absorption, expressed as percentage of the molecule able to pass through the intestinal membrane; [h] prediction of Volume of Distribution (Vd) of the compound in the body; [i] percentage of plasmatic protein bound drug; [j] Ligand affinity toward human serum albumin (HSA); [k] Percentage oral bioavailability.

Table S3. Calculated **1h** ADMET descriptors related to cytochrome inhibition or substrate ability and LD_{50} prediction. Reliability index values for a number of descriptors are shown as R.I. (values higher than 0.30 are ranked as reliable by the software).

**Comp.**	**LD_{50} (mg/kg)**[a] Mouse oral (R.I. ≥ 0.4)	**CYP3A4**[b]		**CYP2D6**[b]	
		**Inhibitor (IC_{50} < 10mM)** (R.I. = 0.14)	**Substrate** (R.I. ≥ 0.3)	**Inhibitor (IC_{50} < 10mM)** (R.I. = 0.38)	**Substrate** (R.I. = 0.23)
**1h**	570	0.52	0.84	0.02	0.03

[a] Acute toxicity (LD_{50}) for mouse after oral administration, [b] Prediction of the ligand inhibitor/substrate behavior towards cytochrome CYP3A4 or CYP2D6.

Figure S1:^{1}H NMR (400 MHz) of compound **1a.**

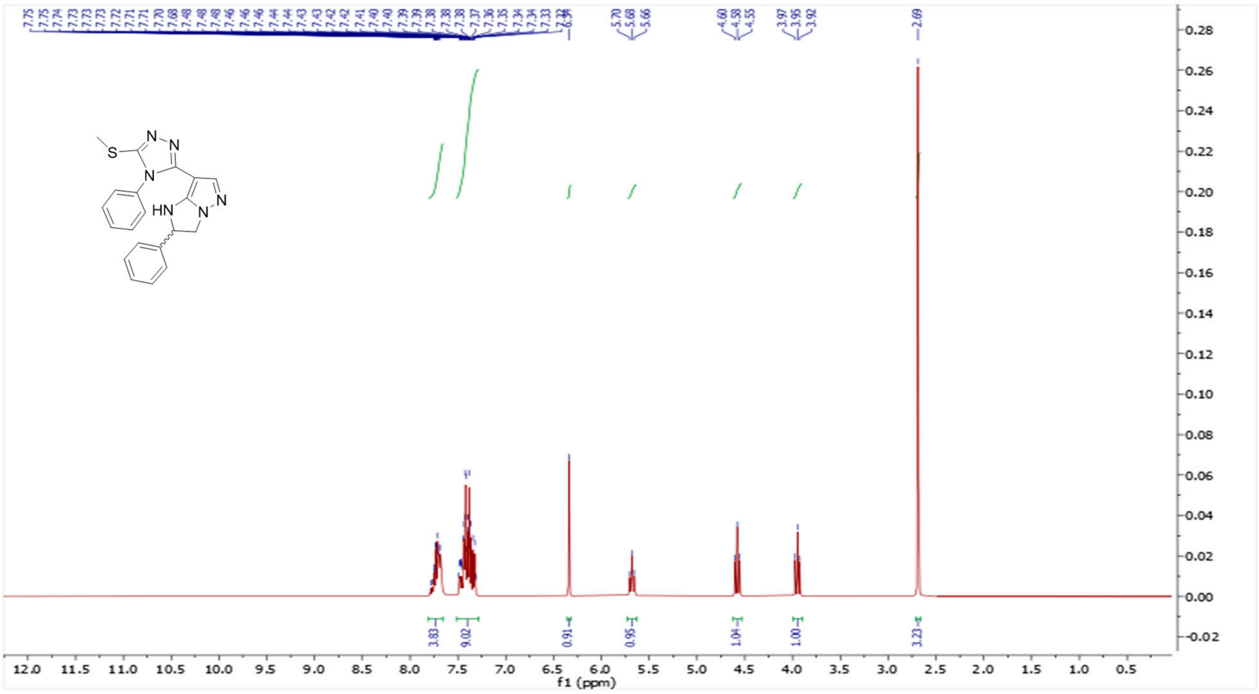

Figure S2: ^{13}C NMR (101 MHz) of compound **1a.**

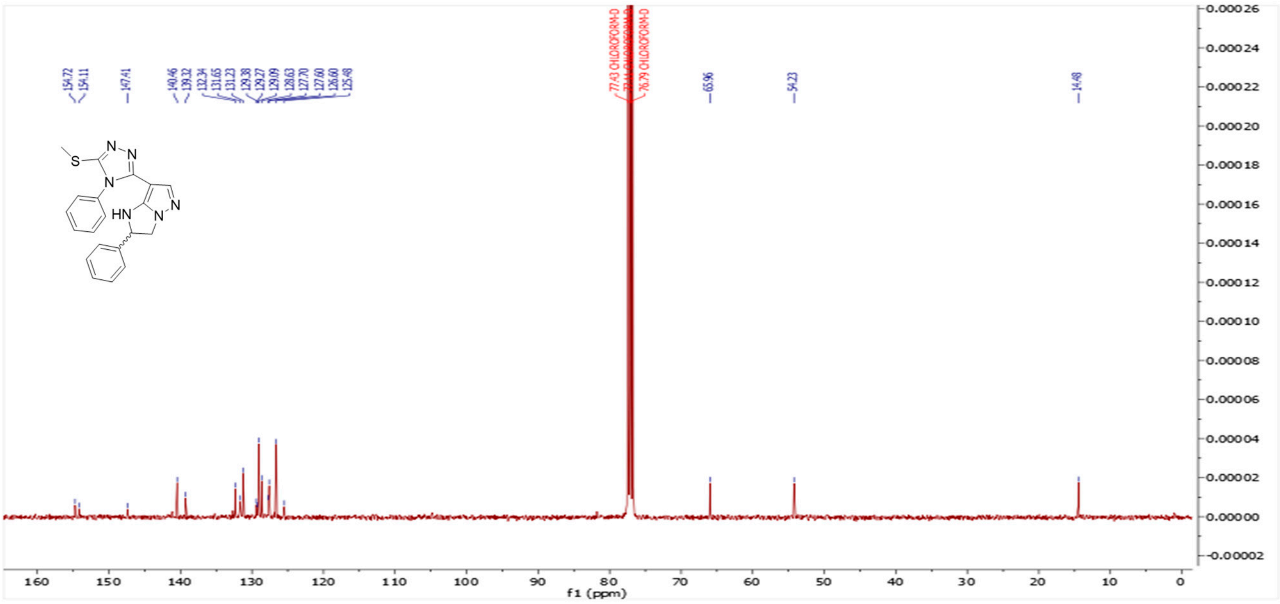

Figure S3: ^{1}H NMR (400 MHz) of compound **1b.**

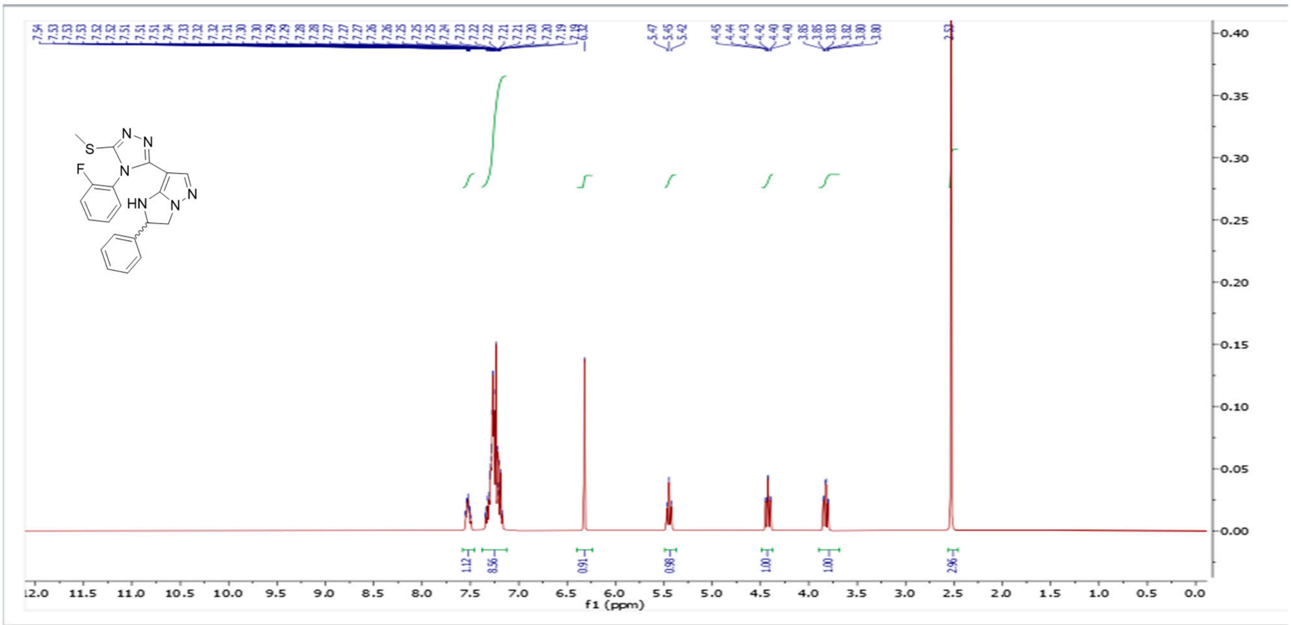

Figure S4: ^{13}C NMR (101 MHz) of compound **1b.**

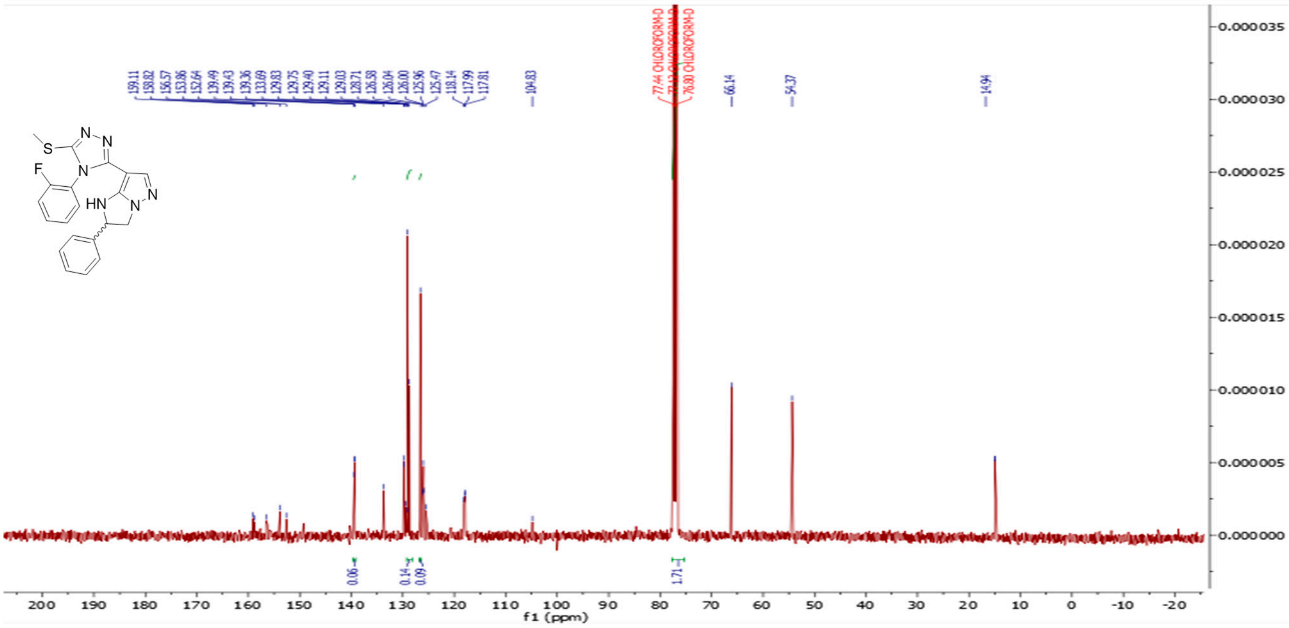

Figure S5:^{1}H NMR (400 MHz) of compound **1c.**

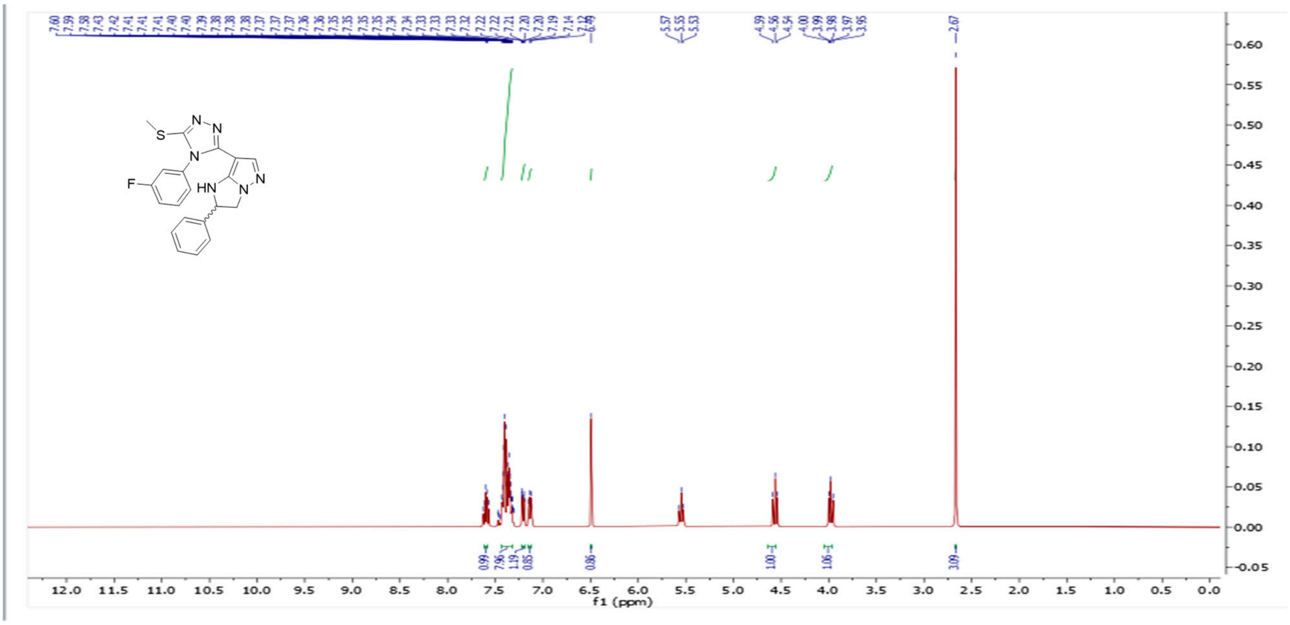

Figure S6: ^{13}C NMR (101 MHz) of compound **1c.**

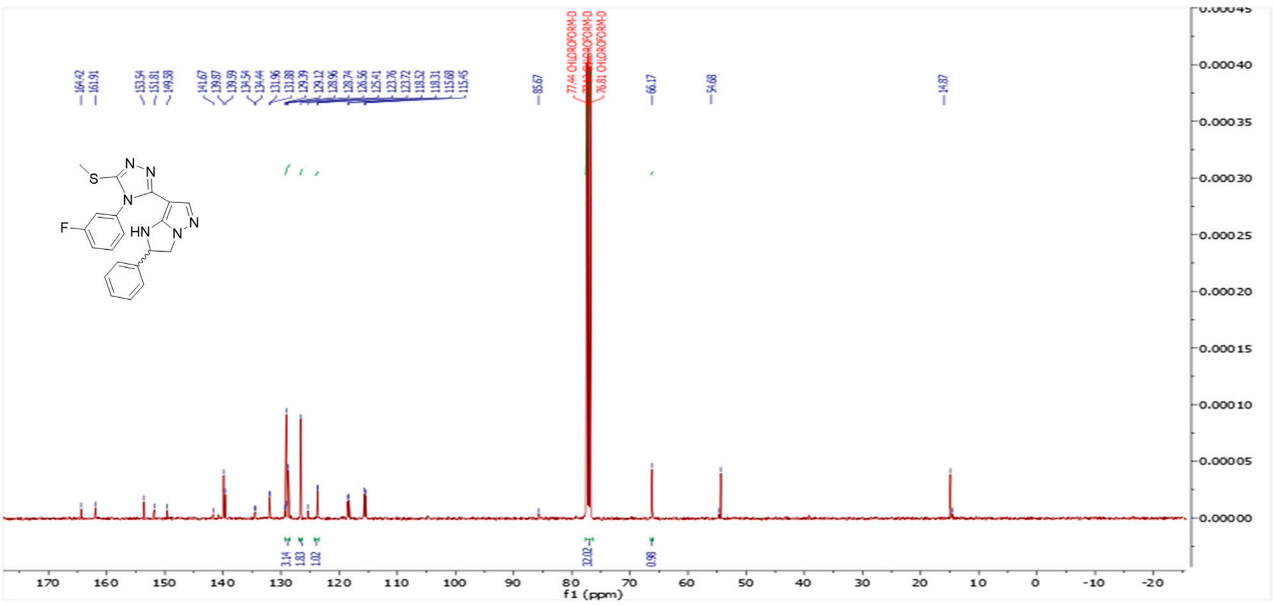

Figure S7:^{1}H NMR (400 MHz) of compound **1d.**

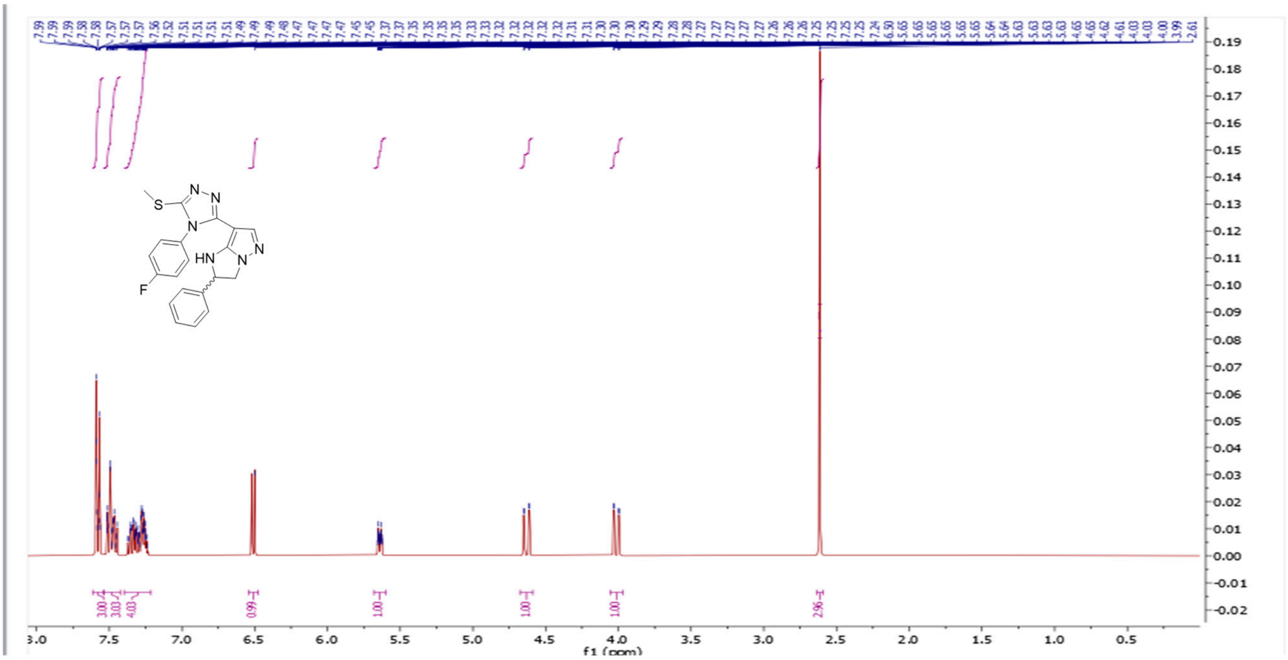

Figure S8: ^{13}C NMR (101 MHz) of compound **1d.**

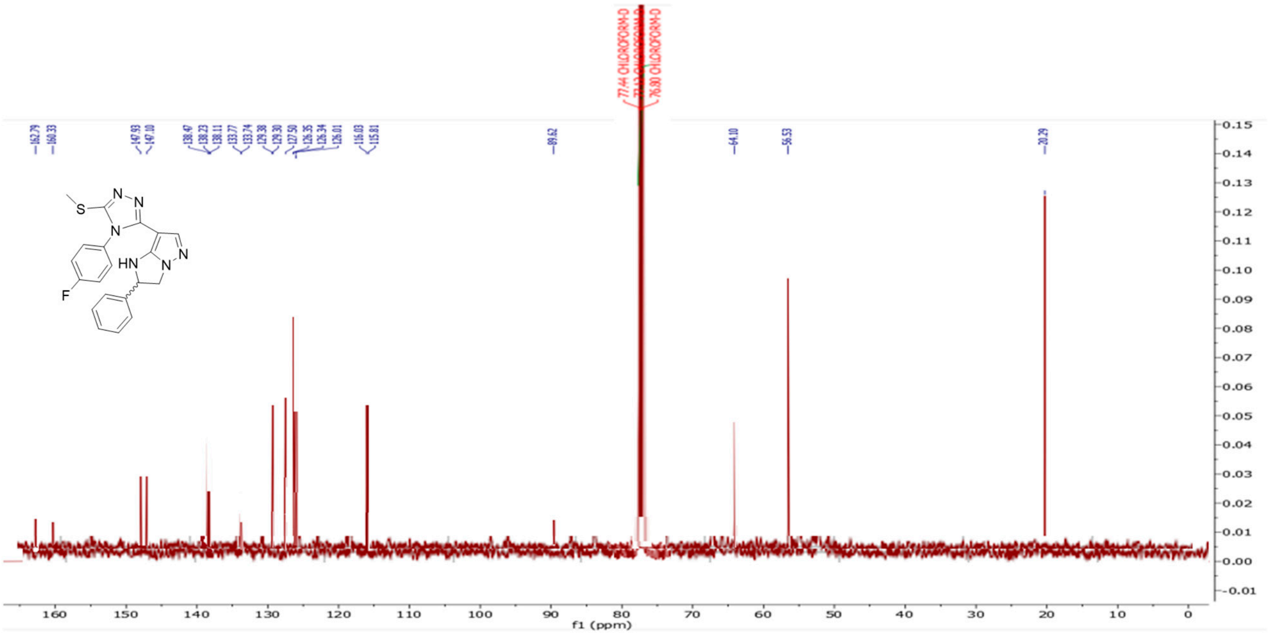

Figure S9: ^{1}H NMR (400 MHz) of compound **1e.**

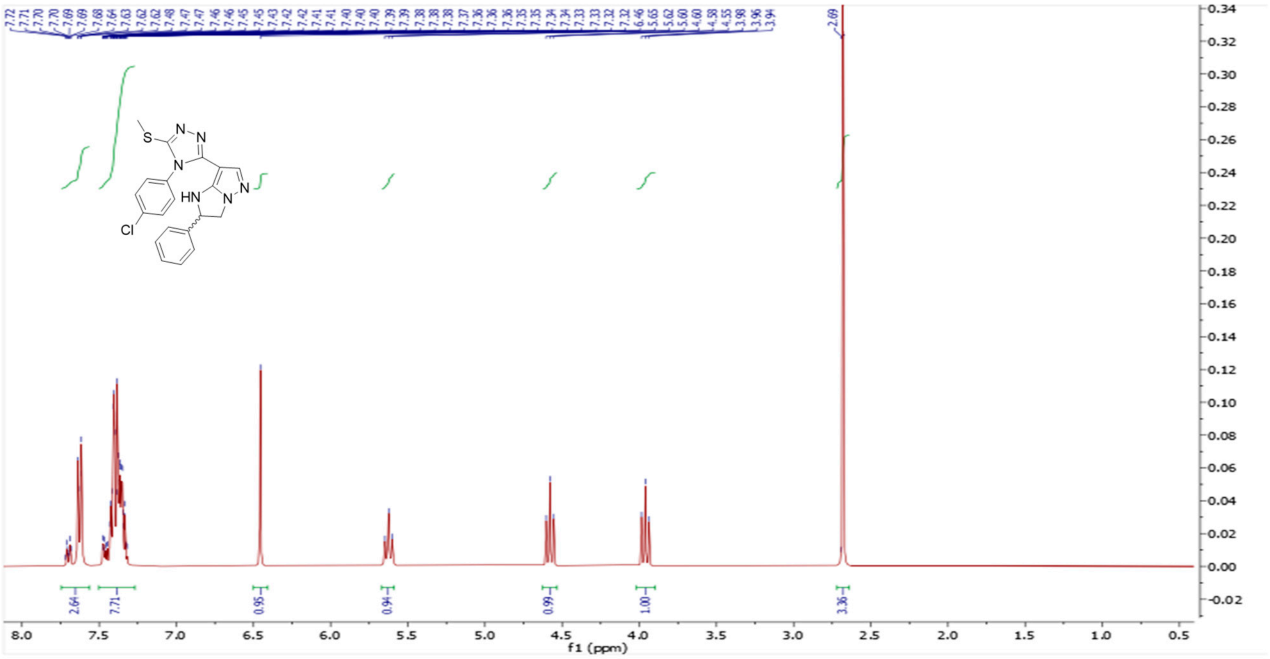

Figure S10: ^{13}C NMR (101 MHz) of compound **1e.**

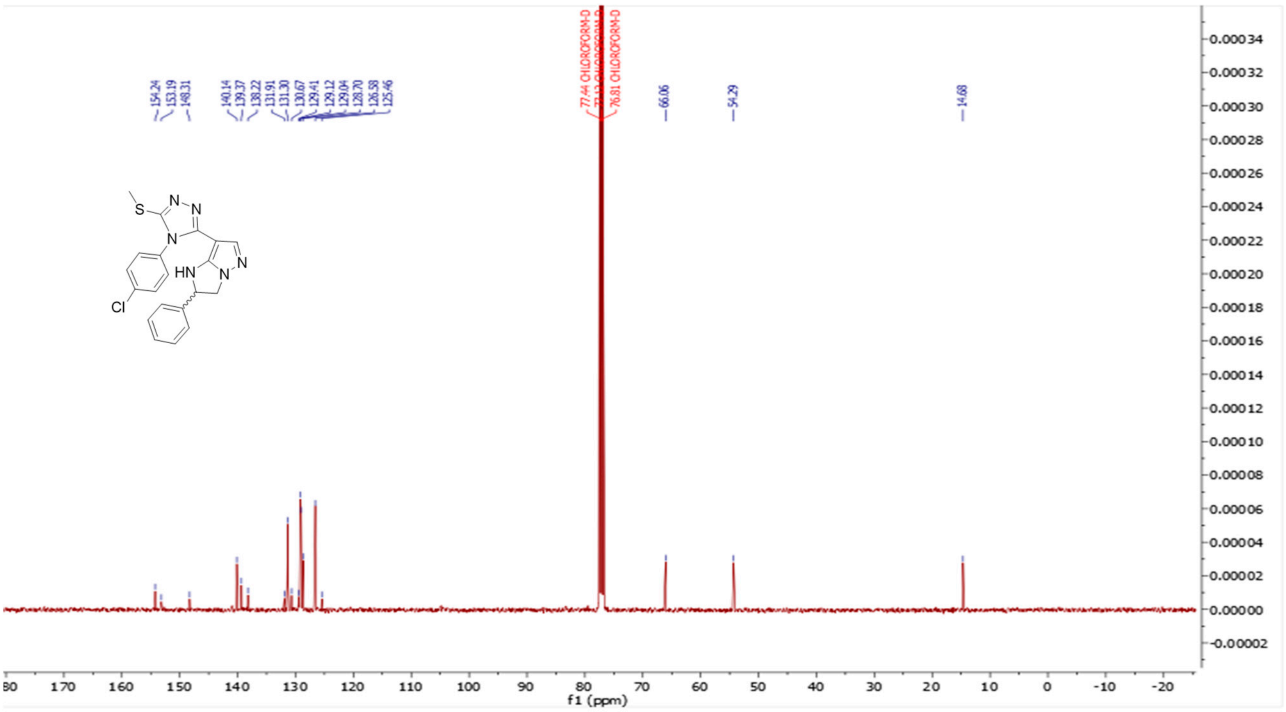

Figure S11: ^{1}H NMR (400 MHz) of compound **1f.**

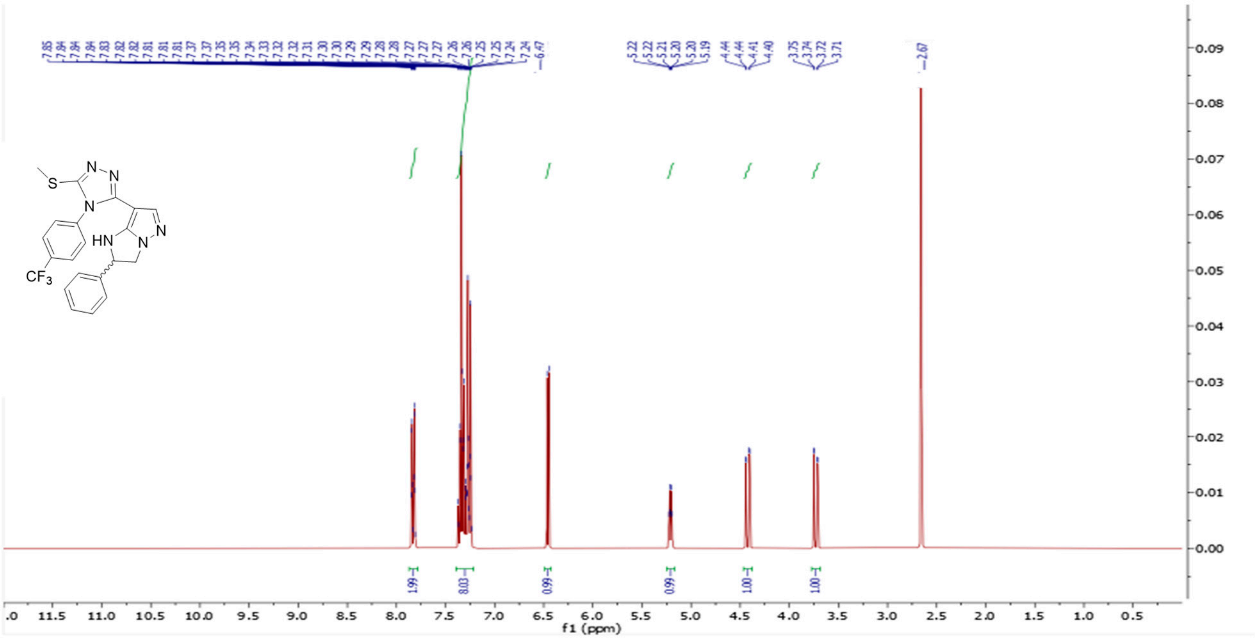

Figure S12: ^{13}C NMR (101 MHz) of compound **1f.**

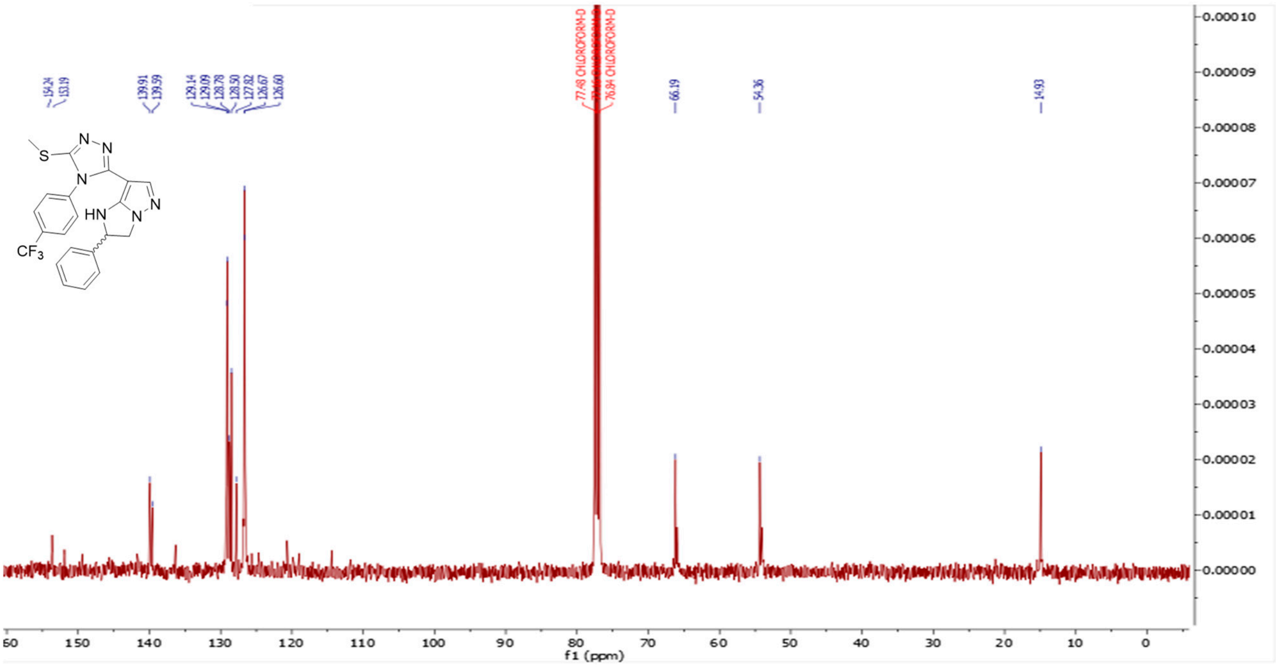

Figure S13: ^{1}H NMR (400 MHz) of compound **1g.**

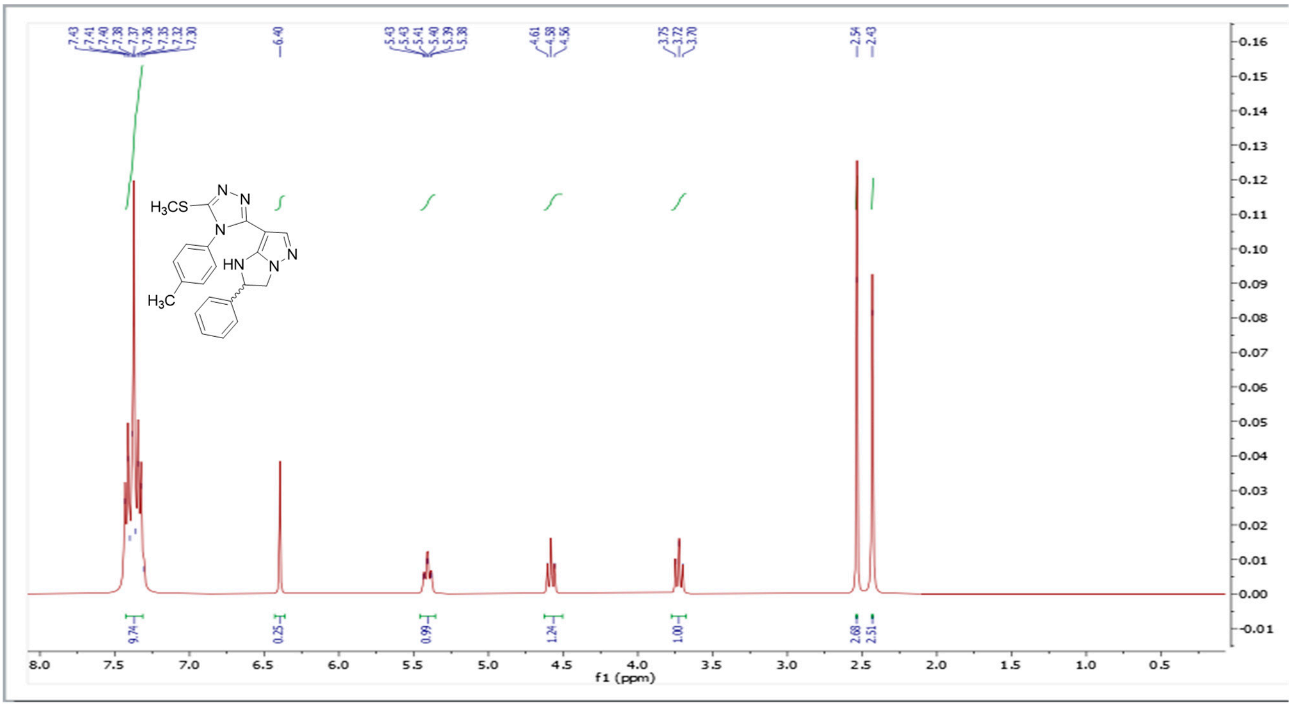

Figure S14: ^{13}C NMR (101 MHz) of compound **1g.**

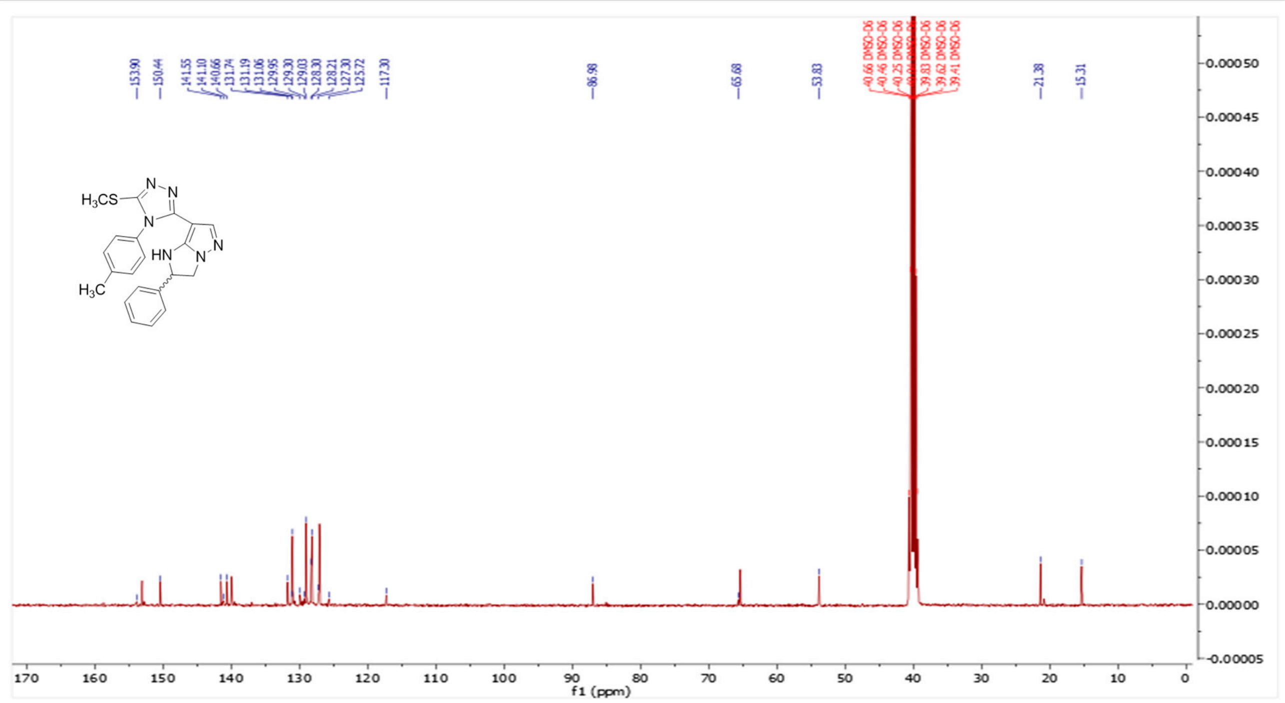

Figure S15:^{1}H NMR (400 MHz) of compound **1h.**

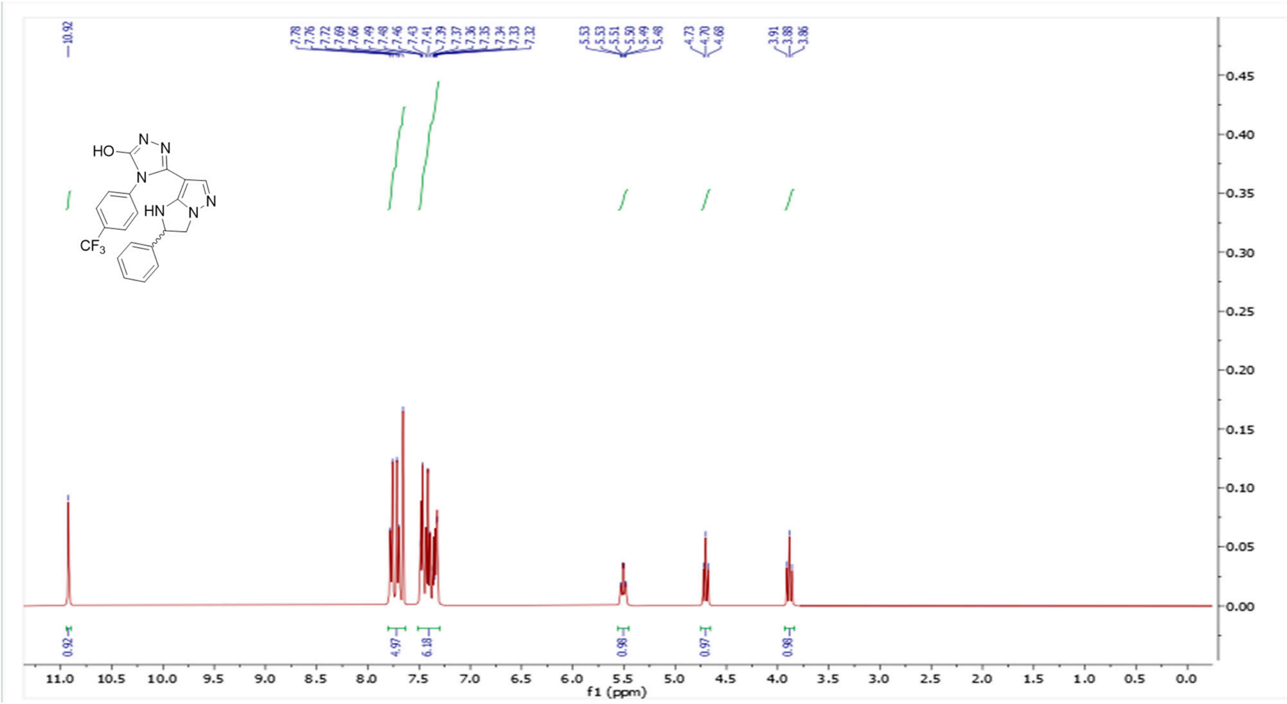

Figure S16: ^{13}C NMR (101 MHz) of compound **1h.**

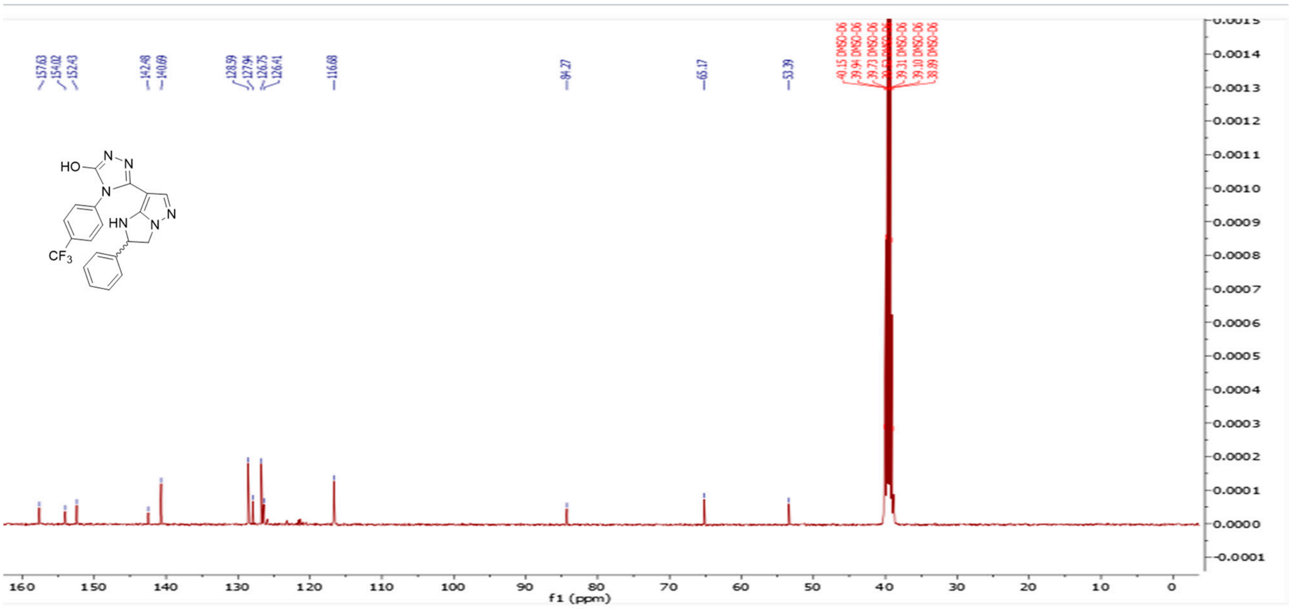

Figure S17: ^{1}H NMR (400 MHz) of compound **4a.**

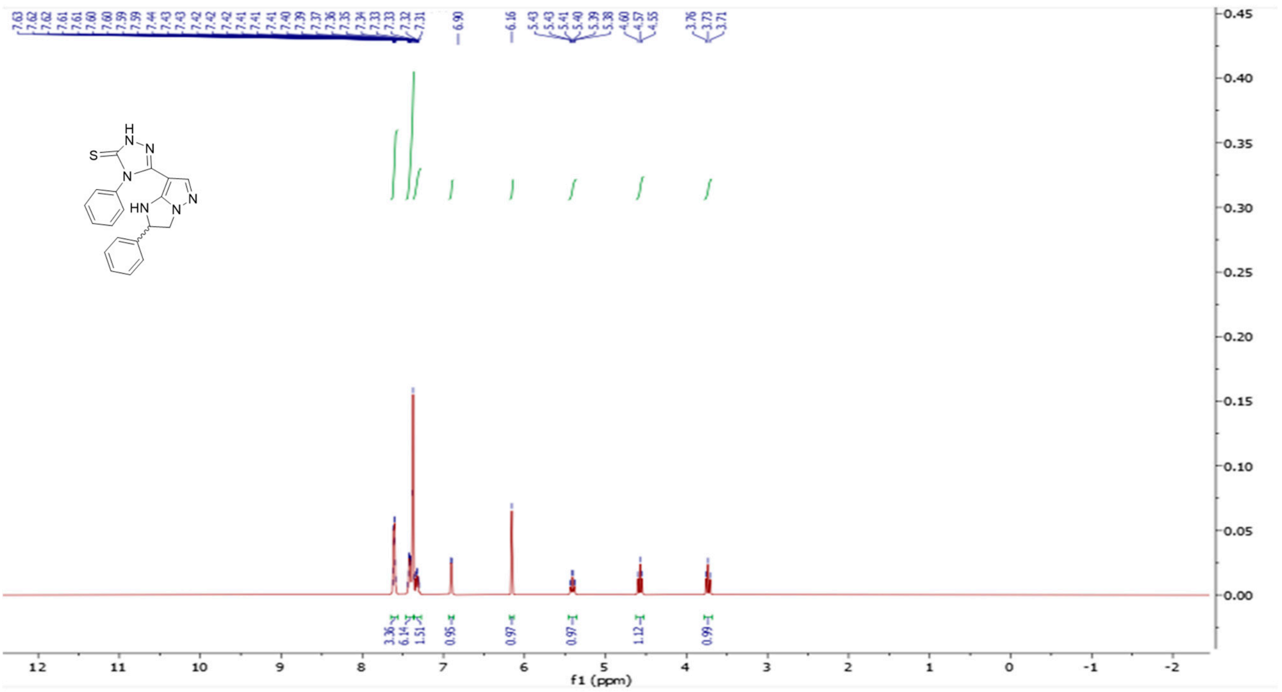

Figure S18: ^{13}C NMR (101 MHz) of compound **4a.**

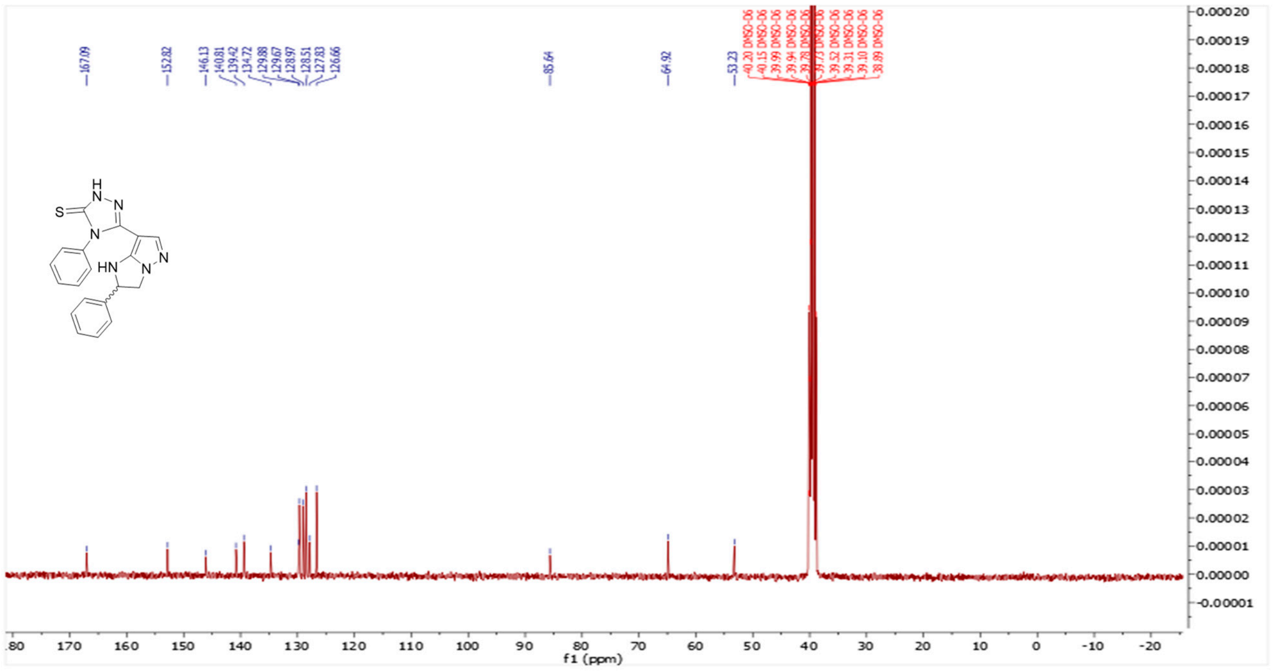

Figure S19:^{1}H NMR (400 MHz) of compound **4b.**

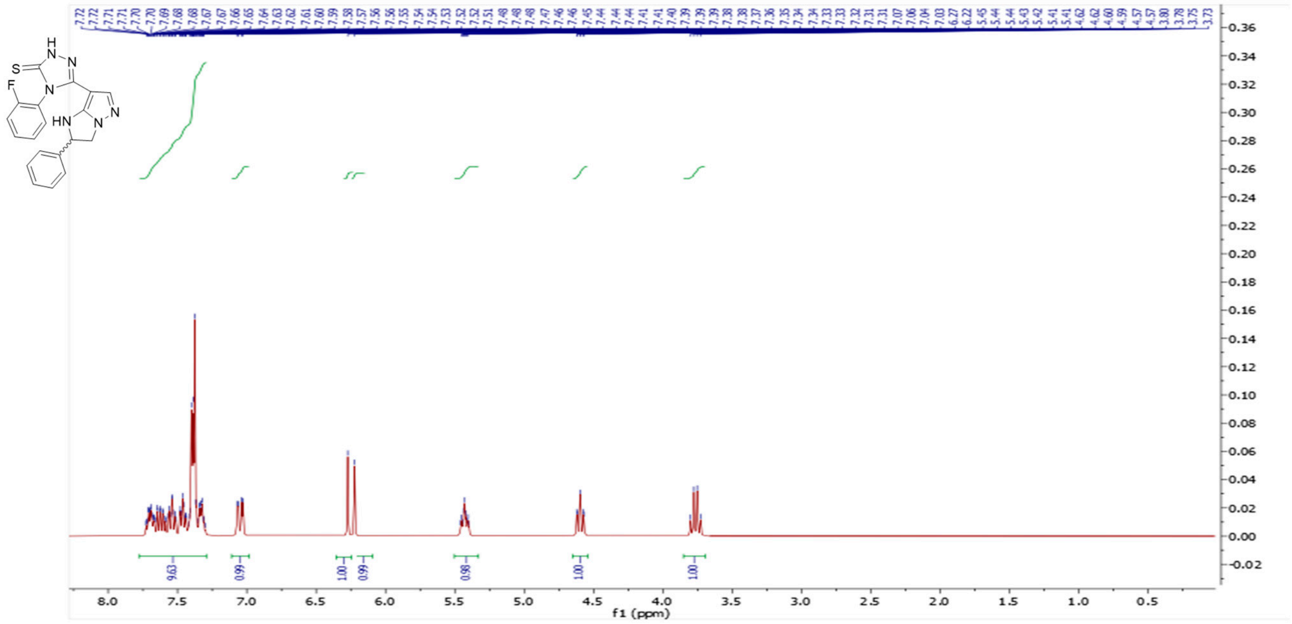

Figure S20: ^{13}C NMR (101 MHz) of compound **4b.**

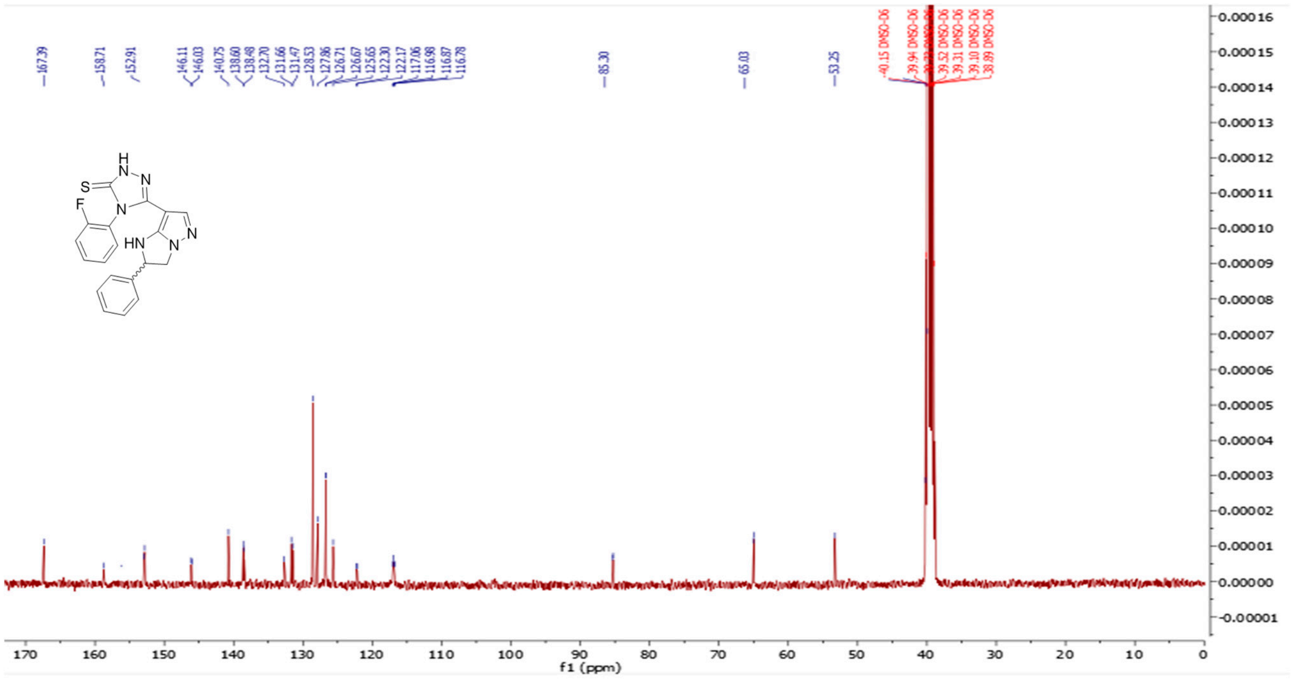

Figure S21:^{1}H NMR (400 MHz) of compound **4c.**

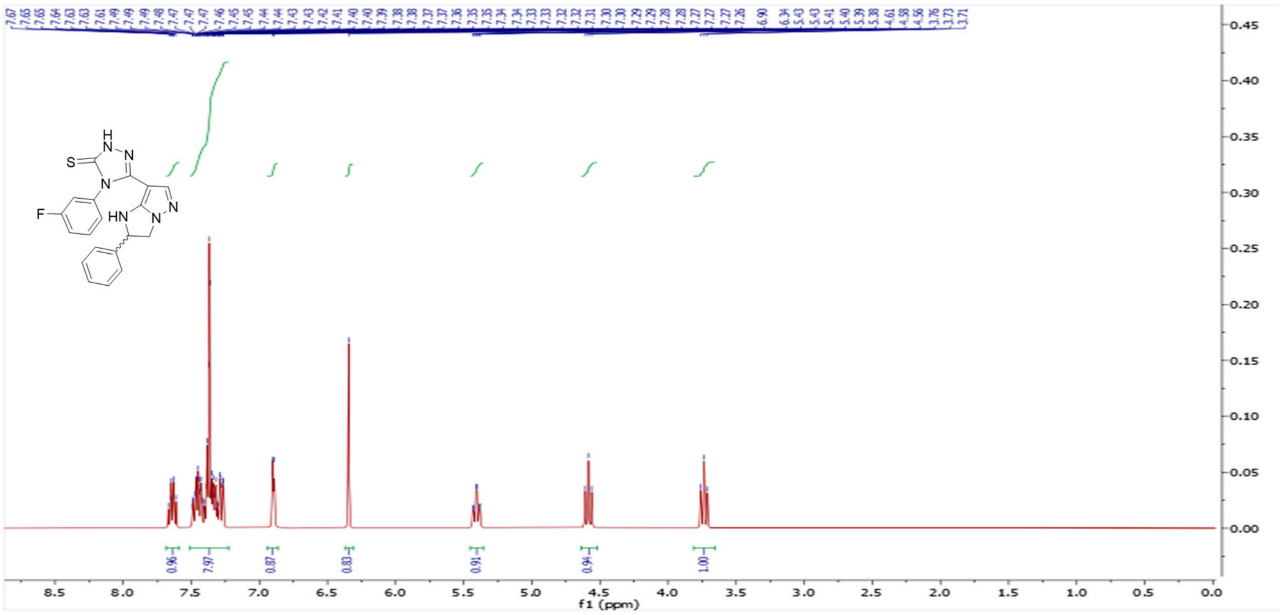

Figure S22: ^{13}C NMR (101 MHz) of compound **4c.**

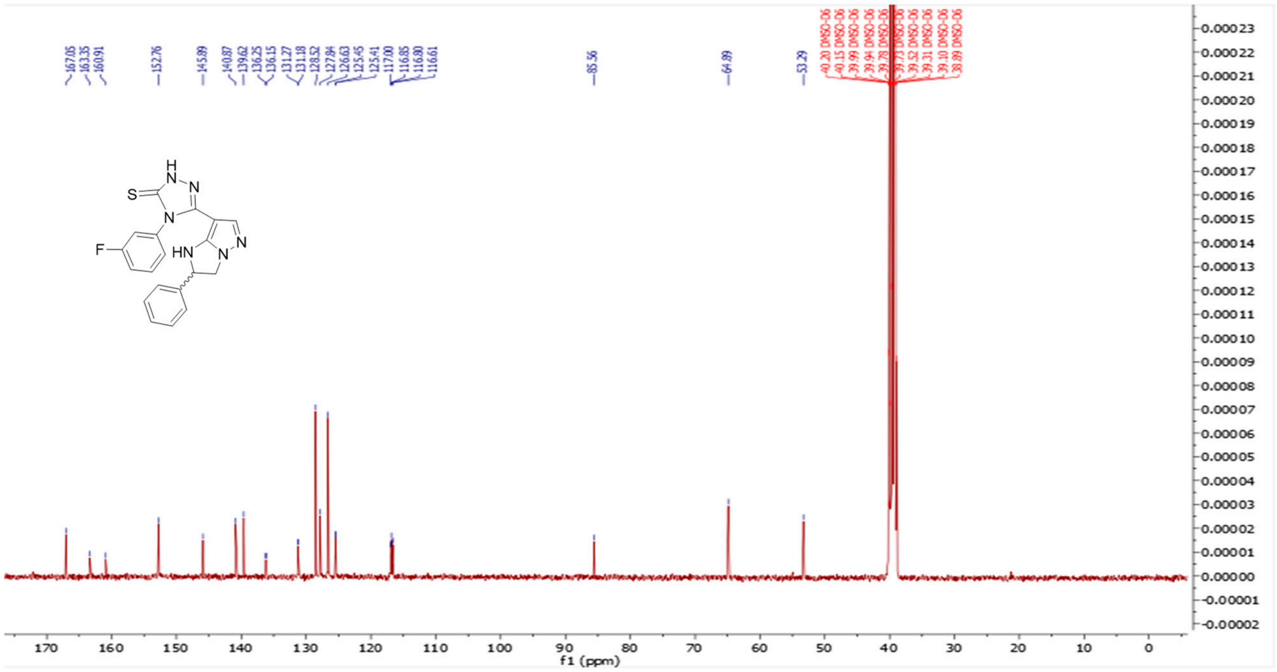

Figure S23:^{1}H NMR (400 MHz) of compound **4d.**

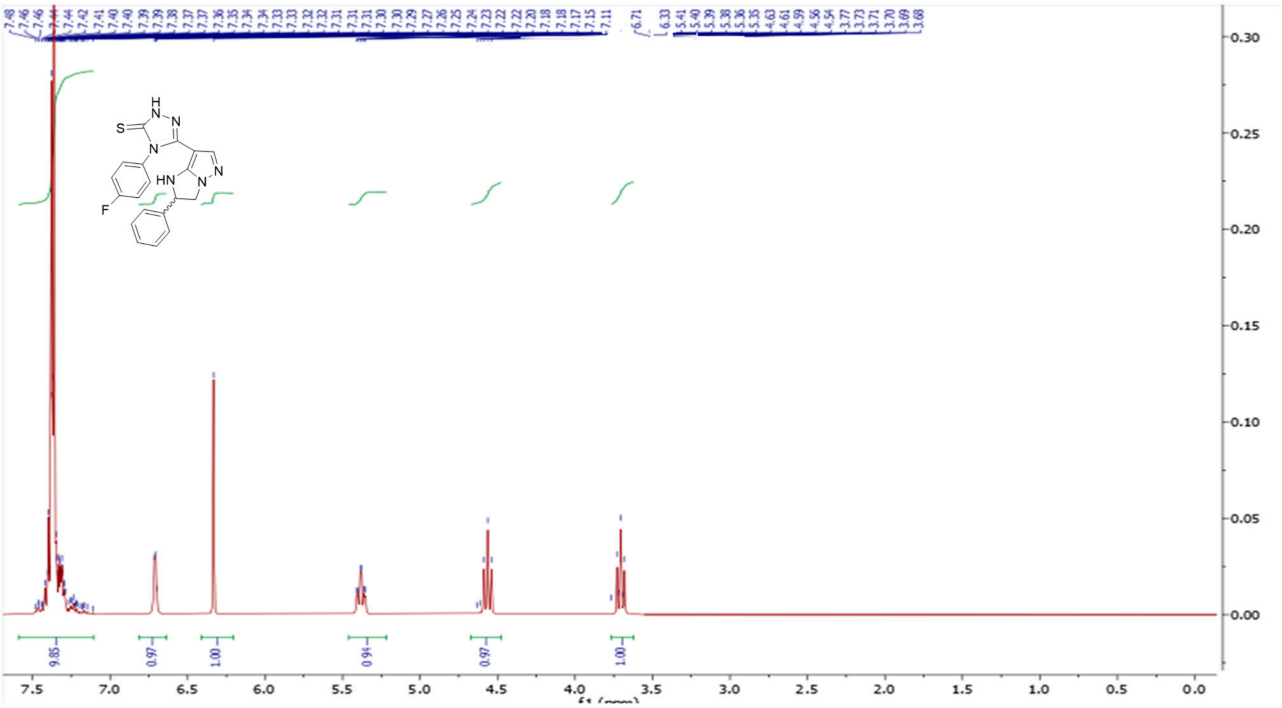

Figure S24: ^{13}C NMR (100 MHz) of compound **4d.**

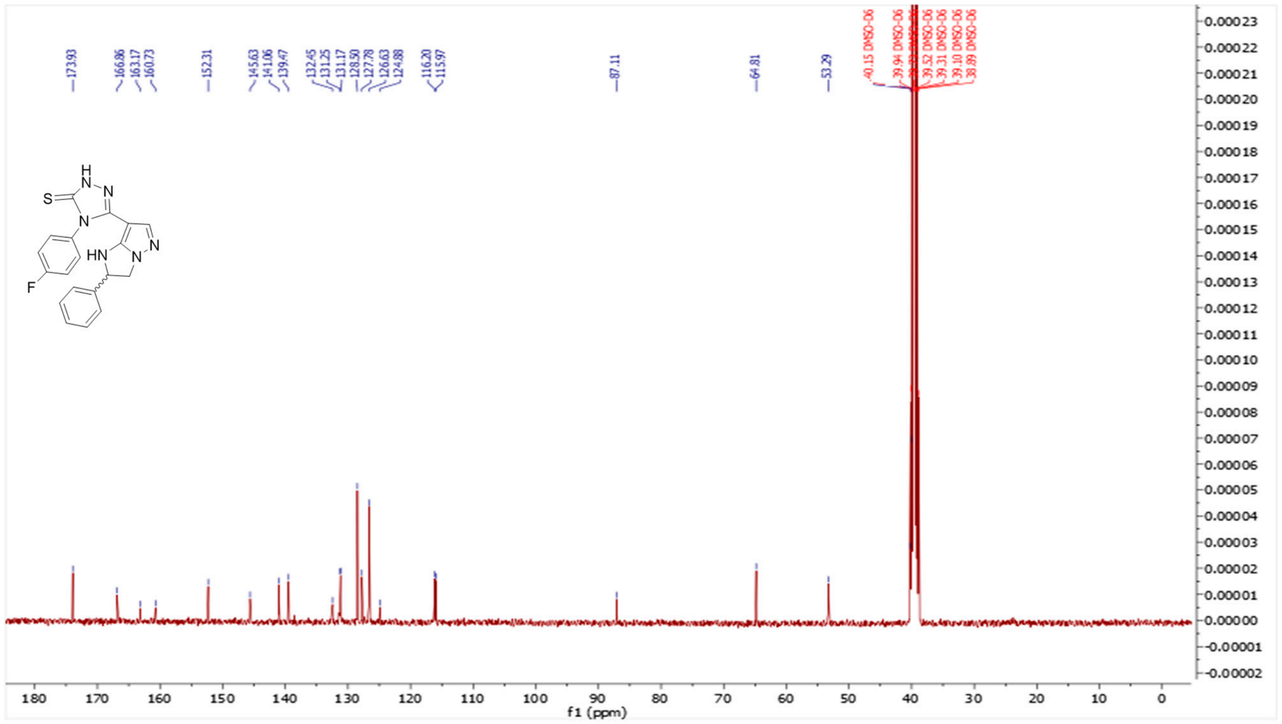

Figure S25:^{1}H NMR (400 MHz) of compound **4e.**

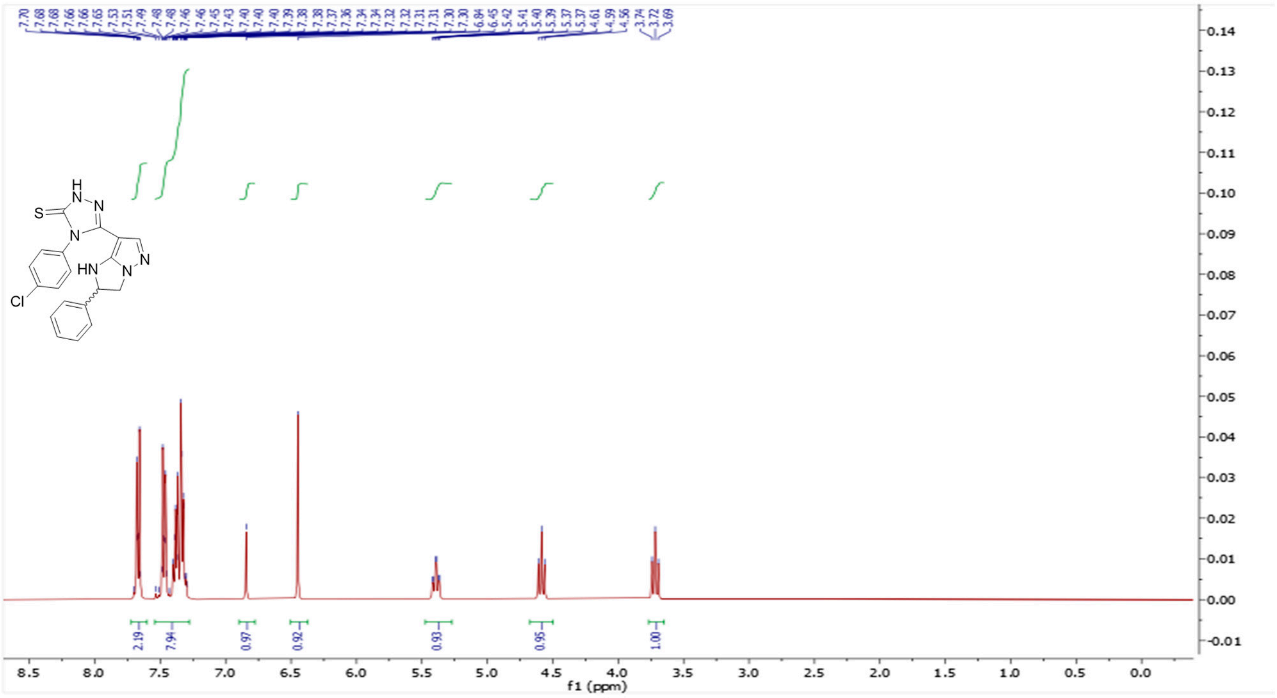

Figure S26: ^{13}C NMR (101 MHz) of compound **4e.**

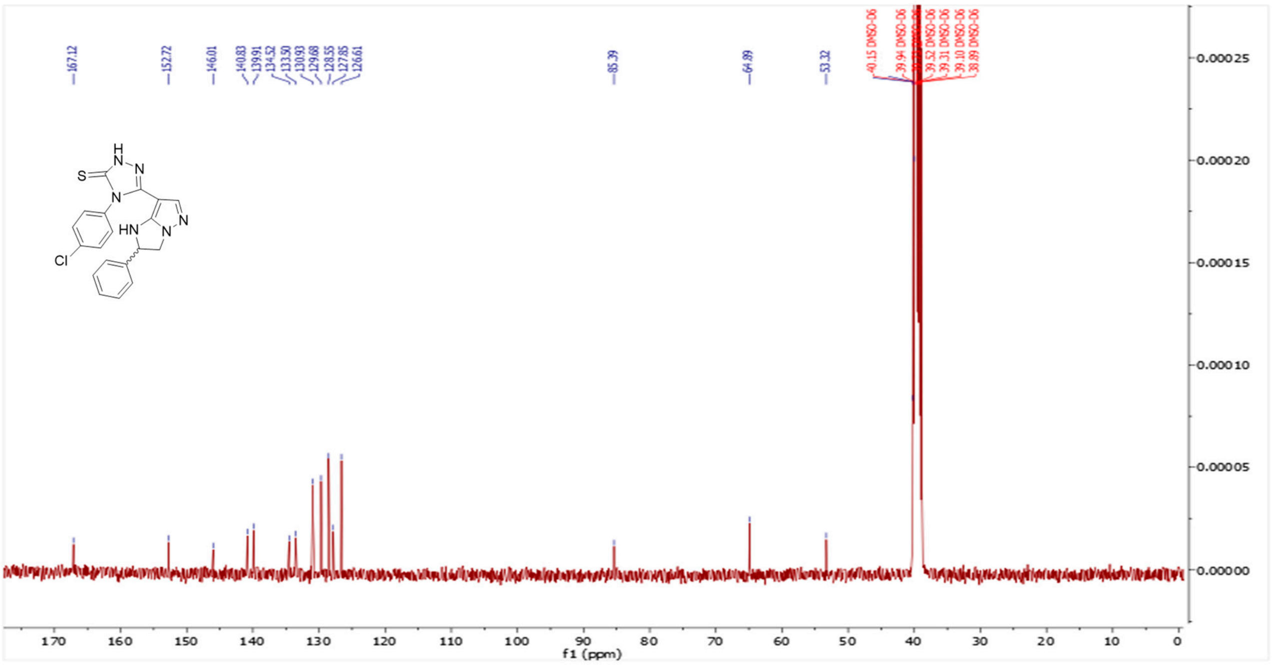

Figure S27: ^{1}H NMR (400 MHz) of compound **4f.**

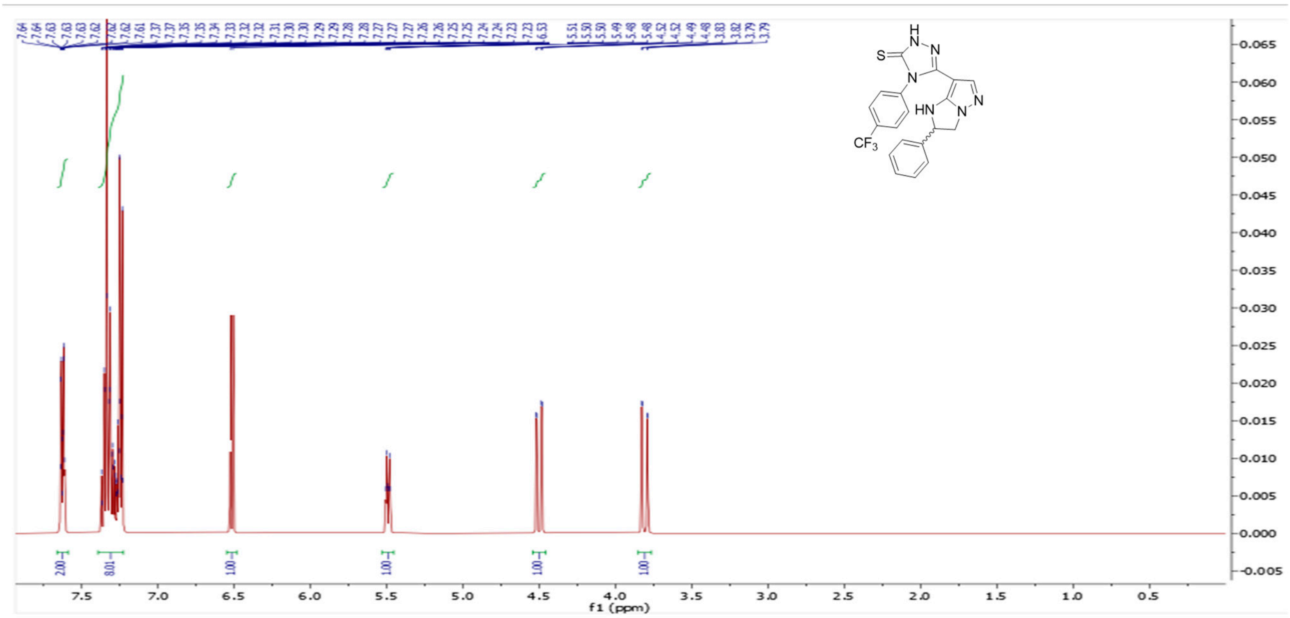

Figure S28: ^{13}C NMR (101 MHz) of compound **4f.**

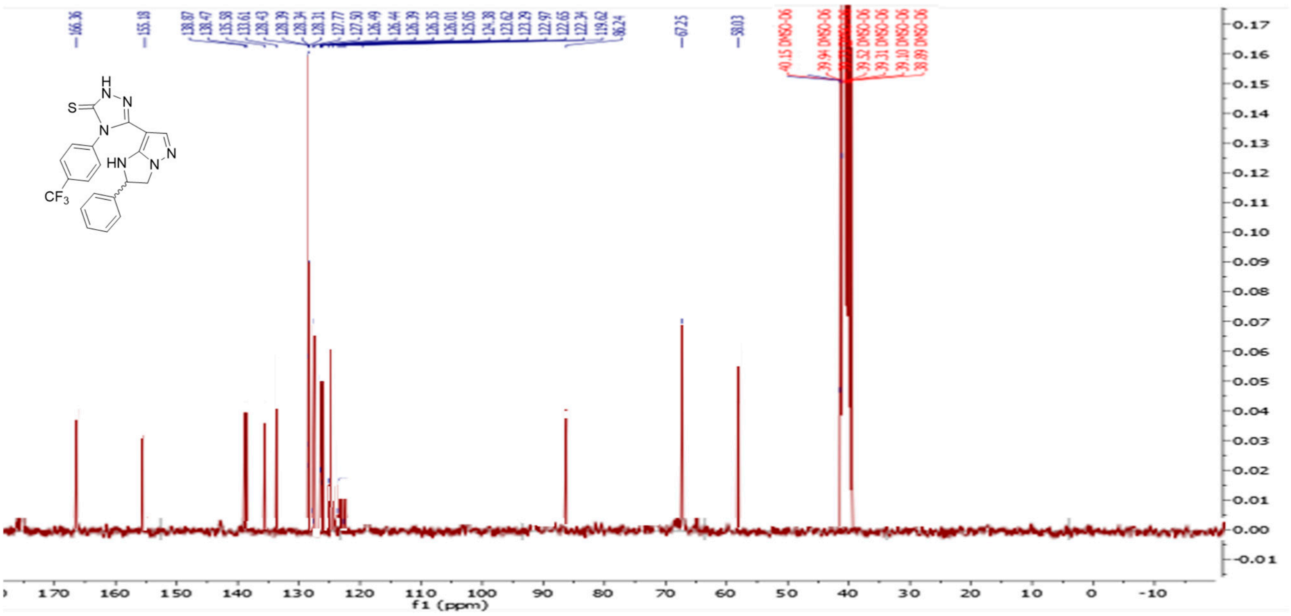

Figure S29: ^{1}H NMR (400 MHz) of compound **4g.**

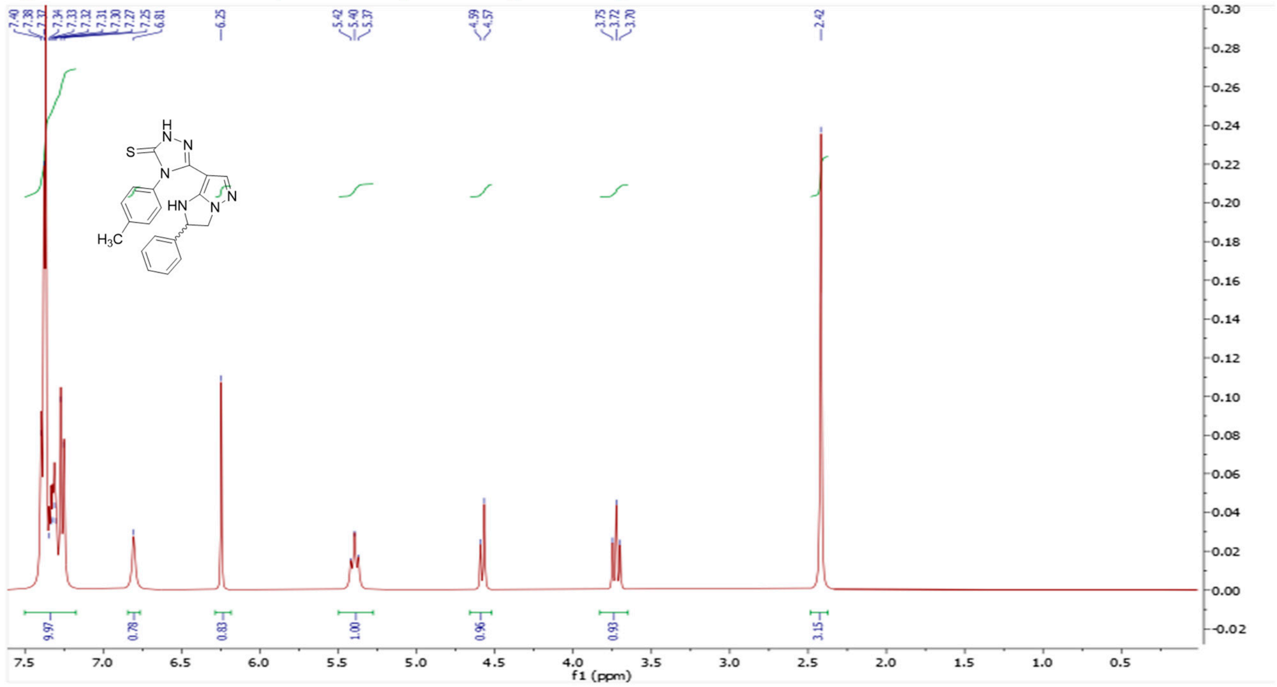

Figure S30: ^{13}C NMR (101 MHz) of compound **4g.**

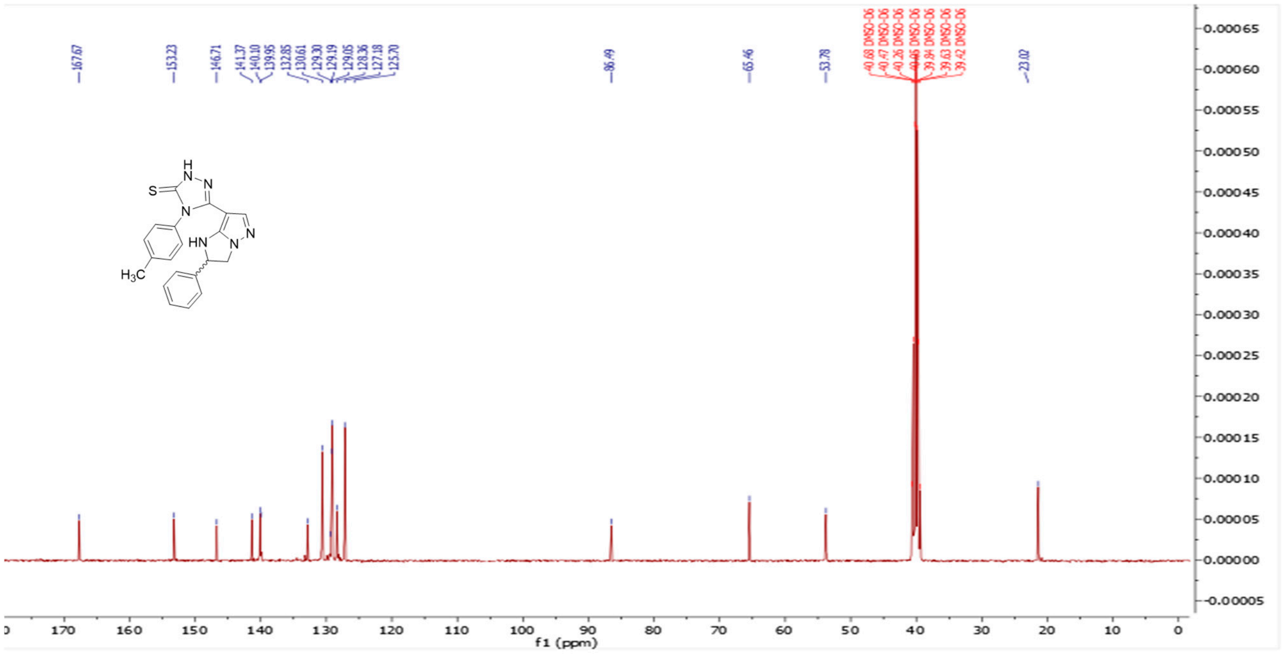

Figure S31: mean graphs of tested of **1a** in one-dose assay

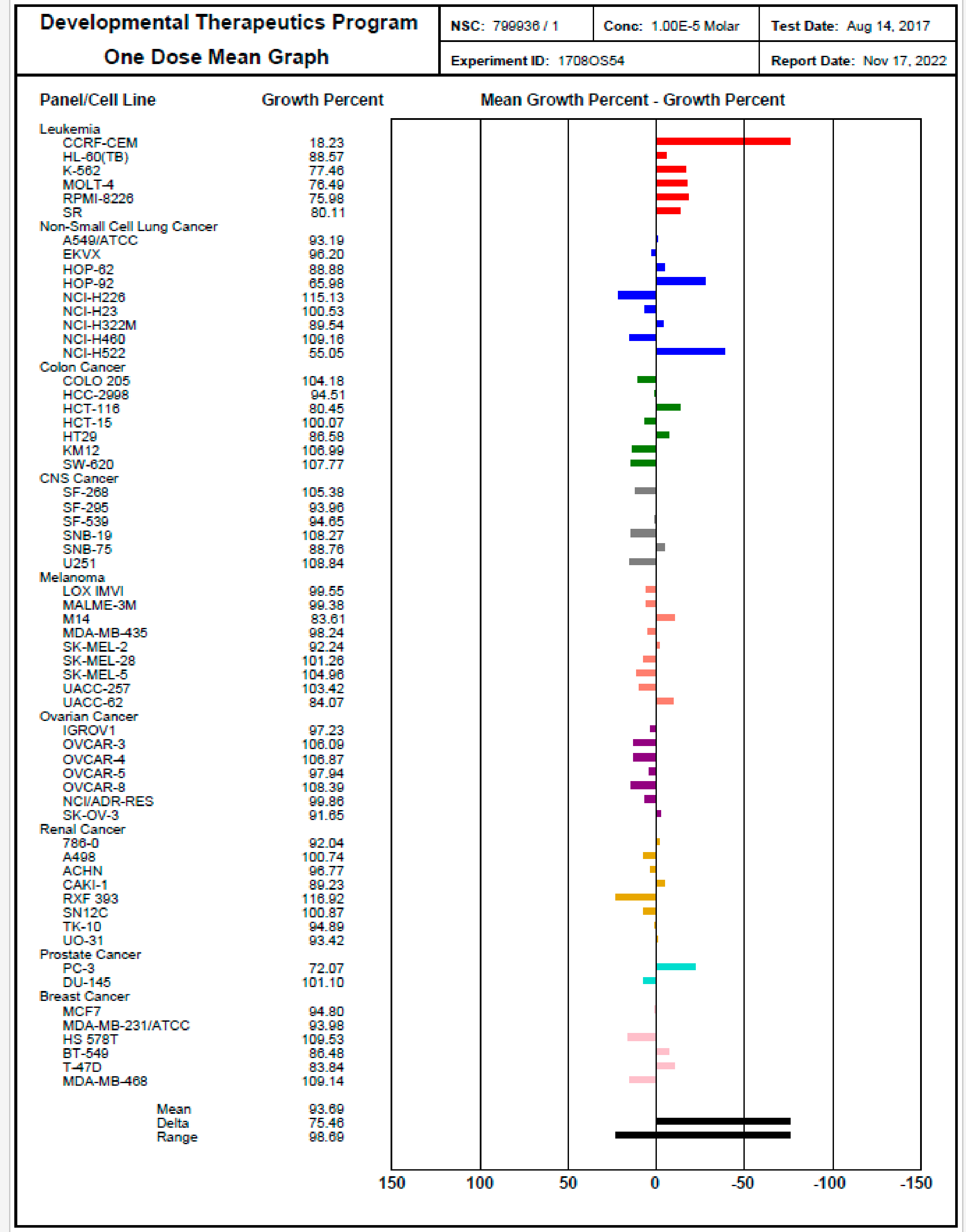

Figure S32: mean graphs of tested of **1e** in one-dose assay

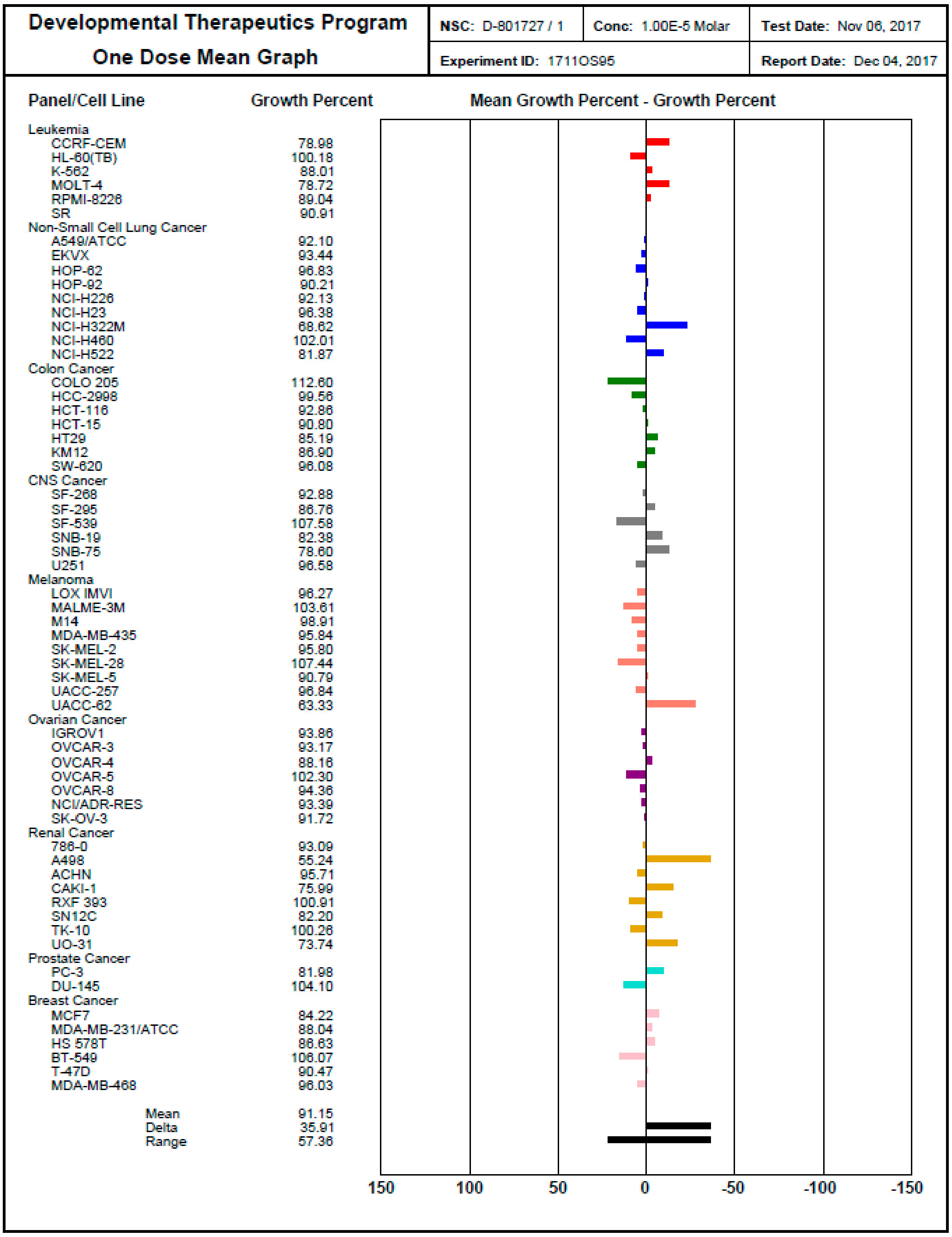

Figure S33: mean graphs of tested of **1h** in one-dose assay

Developmental Therapeutics Program One Dose Mean Graph	NSC: D-801728 / 1	Conc: 1.00E-5 Molar	Test Date: Nov 06, 2017
	Experiment ID: 1711OS95		Report Date: Dec 04, 2017

Panel/Cell Line	Growth Percent
Leukemia	
CCRF-CEM	47.96
HL-60(TB)	73.62
K-562	3.03
MOLT-4	41.87
RPMI-8226	56.88
SR	17.22
Non-Small Cell Lung Cancer	
A549/ATCC	45.49
EKVX	55.99
HOP-62	61.10
HOP-92	64.30
NCI-H226	73.54
NCI-H23	72.64
NCI-H322M	72.27
NCI-H460	61.84
NCI-H522	26.08
Colon Cancer	
COLO 205	63.92
HCC-2998	85.86
HCT-116	46.00
HCT-15	35.08
HT29	28.39
KM12	49.25
SW-620	50.88
CNS Cancer	
SF-268	65.13
SF-295	32.01
SF-539	80.34
SNB-19	66.88
SNB-75	66.68
U251	58.04
Melanoma	
LOX IMVI	61.26
MALME-3M	69.79
M14	54.32
MDA-MB-435	-25.66
SK-MEL-2	50.21
SK-MEL-28	71.67
SK-MEL-5	31.31
UACC-257	51.37
UACC-62	40.27
Ovarian Cancer	
IGROV1	69.82
OVCAR-3	37.58
OVCAR-4	59.09
OVCAR-5	84.67
OVCAR-8	79.39
NCI/ADR-RES	56.01
SK-OV-3	69.36
Renal Cancer	
786-0	66.09
A498	46.80
ACHN	52.27
CAKI-1	32.04
RXF 393	79.08
SN12C	78.54
TK-10	70.57
UO-31	53.70
Prostate Cancer	
PC-3	46.28
DU-145	85.07
Breast Cancer	
MCF7	26.87
MDA-MB-231/ATCC	64.76
HS 578T	64.72
BT-549	85.71
T-47D	54.23
MDA-MB-468	71.08
Mean	55.68
Delta	81.34
Range	111.52

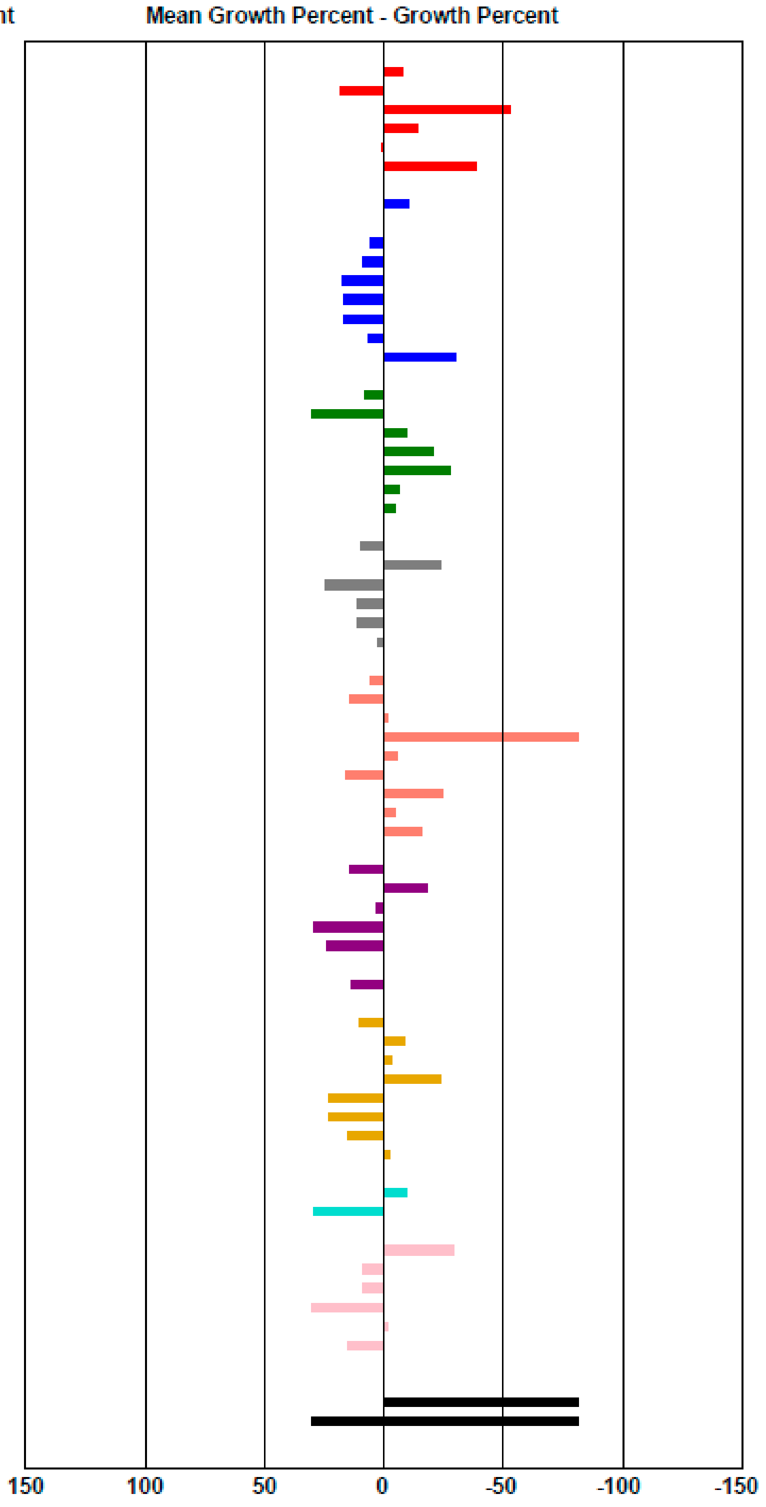

Supplement: Supplementary file 1 [file ijms-26-06312-s001.zip › ijms-3688419-supplementary.pdf]
